# Repurposing of lonafarnib as a treatment for SARS-CoV-2 infection

**DOI:** 10.1172/jci.insight.182704

**Published:** 2025-01-09

**Authors:** Mohsin Khan, Parker Irvin, Seung Bum Park, Hannah M. Ivester, Inna Ricardo-Lax, Madeleine Leek, Ailis Grieshaber, Eun Sun Jang, Sheryl Coutermarsh-Ott, Qi Zhang, Nunziata Maio, Jian-Kang Jiang, Bing Li, Wenwei Huang, Amy Q. Wang, Xin Xu, Zongyi Hu, Wei Zheng, Yihong Ye, Tracey Rouault, Charles Rice, Irving C. Allen, T. Jake Liang

**Affiliations:** 1 Liver Diseases Branch, National Institute of Diabetes and Digestive and Kidney Diseases, NIH, Bethesda, Maryland, USA.; 2 Department of Biomedical Sciences and Pathobiology, Virginia-Maryland College of Veterinary Medicine, Virginia Tech, Blacksburg, Virginia, USA.; 3 Laboratory of Virology and Infectious Disease, The Rockefeller University, New York, New York, USA.; 4 Division of Preclinical Innovation, National Center for Advancing Translational Sciences, NIH, Rockville, Maryland, USA.; 5 Eunice Kennedy Shriver National Institute of Child Health and Human Development, NIH, Bethesda, Maryland, USA.; 6 Laboratory of Molecular Biology, National Institute of Diabetes and Digestive and Kidney Diseases, NIH, Bethesda, Maryland, USA.

**Keywords:** COVID-19, Virology, Drug screens, Drug therapy, Molecular biology

## Abstract

Severe acute respiratory syndrome coronavirus 2 (SARS-CoV-2), which causes coronavirus disease 2019 (COVID-19), has emerged as a global pandemic pathogen with high mortality. While treatments have been developed to reduce morbidity and mortality of COVID-19, more antivirals with broad-spectrum activities are still needed. Here, we identified lonafarnib (LNF), a Food and Drug Administration–approved inhibitor of cellular farnesyltransferase (FTase), as an effective anti–SARS-CoV-2 agent. LNF inhibited SARS-CoV-2 infection and acted synergistically with known anti-SARS antivirals. LNF was equally active against diverse SARS-CoV-2 variants. Mechanistic studies suggested that LNF targeted multiple steps of the viral life cycle. Using other structurally diverse FTase inhibitors and a LNF-resistant FTase mutant, we demonstrated a key role of FTase in the SARS-CoV-2 life cycle. To demonstrate in vivo efficacy, we infected SARS-CoV-2–susceptible humanized mice expressing human angiotensin-converting enzyme 2 (ACE2) and treated them with LNF. LNF at a clinically relevant dose suppressed the viral titer in the respiratory tract and improved pulmonary pathology and clinical parameters. Our study demonstrated that LNF, an approved oral drug with excellent human safety data, is a promising antiviral against SARS-CoV-2 that warrants further clinical assessment for treatment of COVID-19 and potentially other viral infections.

## Abstract 2

Lonafarnib, an FDA-approved drug, is a potent antiviral against SAS-CoV-2 and can be developed as a promising drug for the treatment of COVID-19 and other viral infections.

## Graphical abstract

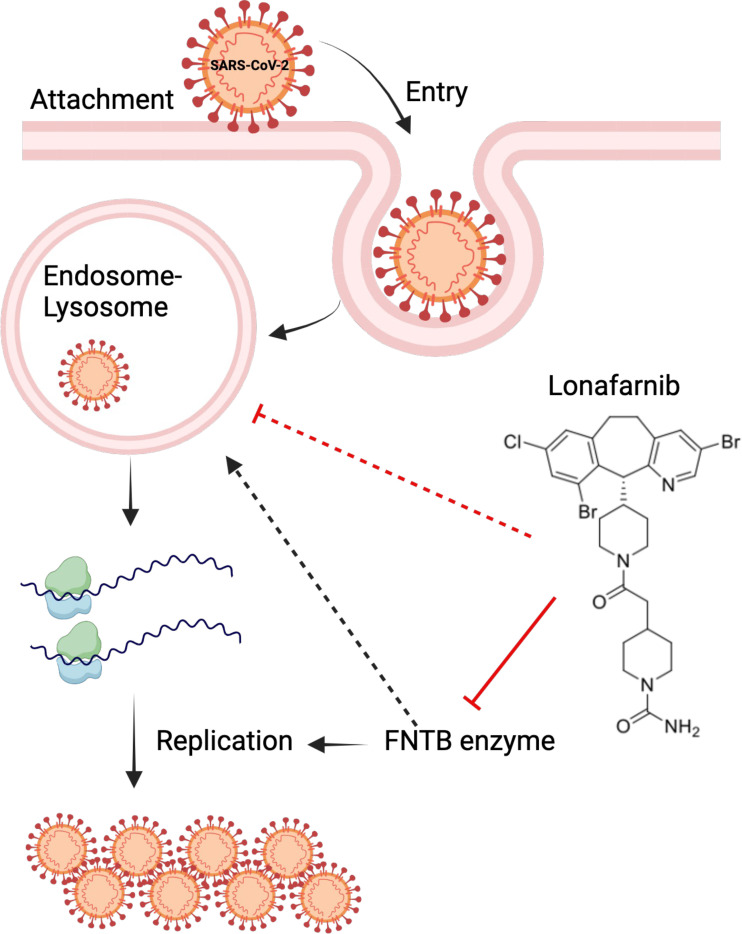

## Introduction

Severe acute respiratory syndrome coronavirus 2 (SARS-CoV-2) is a positive-sense single-stranded-RNA virus (1). The genomic RNA requires RNA-dependent RNA polymerase (RdRp) for replication. The genome is approximately 30 kb long and encodes 16 genes with various functions required for productive infection (2, 3). The viral glycoprotein of SARS-CoV-2 (spike, S) is cleaved by furin proteases and produces 2 functional domains, S1 and S2, which mediate receptor binding and membrane fusion respectively (2). The interaction between angiotensin-converting enzyme 2 (ACE2) and S2 results in cleavage of S protein by cellular proteases like transmembrane protease serine subtype 2 (TMPRSS2) (4, 5). This cleavage then facilitates membrane fusion that ensures the successful delivery of genomic RNA into the cells. In addition, SARS-CoV-2 can enter the cell via a receptor-mediated endocytosis pathway, which is mainly mediated by ACE2 and a pH-dependent process (6, 7).

Therapeutic development against SARS-CoV-2 has been an intensely active area of research since the onset of COVID-19 and has led to multiple modalities of treatment options (8–10). Multiple direct-acting antivirals (DAAs) have been developed to target various steps of the SARS-CoV-2 life cycle (11, 12). Only a few effective antiviral drugs against COVID-19 have been approved by the FDA. Remdesivir (RDV), a nucleotide analog, was shown to be effective in earlier clinical trials and thus the first approved drug for COVID-19. Subsequently, a large trial showed that RDV had limited benefits in COVID-19 patients, such as those with mild to moderate symptoms (13–16).

As second-generation DAAs, Paxlovid, a protease inhibitor (nirmatrelvir, NRTV) in combination with ritonavir, and Lagevrio, a nucleoside analog (molnupiravir) received emergency use authorization from the FDA in early 2022 (17–20). Both drugs are not authorized for patients requiring hospitalization due to severe or critical COVID-19, for certain age groups, for longer than 5 consecutive days of treatment, nor for pre-exposure or post-exposure prophylaxis. Moreover, viral rebound and disease relapse have been reported not infrequently in Paxlovid-treated patients (15, 21). A recent large randomized-control study did not indicate any clinical benefits of Paxlovid in vaccinated or unvaccinated adult outpatients without increased risks of severe COVID-19 (22). Monoclonal antibodies targeting the S envelope protein of SARS-CoV-2 capable of preventing viral entry have been developed and shown effective in ameliorating COVID-19 disease in earlier clinical studies (23). But they are less effective against the newly emerged variants due to spike mutations (24).

Drug repurposing, in which approved drugs are tested for treatment of diseases other than their original indication, offers many advantages over conventional drug development. Since repurposed drugs have already been found to be safe and gone through extensive clinical testing, risks of safety failure are low and development timeline can be fast-tracked (25). Previously, we successfully identified multiple hepatitis C virus (HCV) inhibitors that target early events of the viral life cycle. These compounds included both new chemical entities and previously known pharmaceutical compounds. Many of those drugs were antihistamines (26–30). Notably, the cellular events of the early viral life cycle such as endocytosis and membrane fusion are relatively conserved among diverse viral families (31, 32). We tested a number of these compounds against SARS-CoV-2 and demonstrated antiviral activity that also targets viral fusion (33). To further explore the feasibility of developing potent anti–SARS-CoV-2 drugs based on this mechanism, we screened additional functionally and structurally related compounds. We identified lonafarnib (LNF) as a potential anti–SARS-CoV-2 compound. We also tested RDV and NRTV and found that both drugs exert a synergistic effect when used in combination with LNF. Finally, we demonstrated that LNF treatment reduced the viral titer and disease severity in a mouse model of SARS-CoV-2 infection. Taken together, our results provide a solid platform for LNF to be further investigated as an anti–SARS-CoV-2 drug and demonstrate that cellular farnesyltransferase is a promising host target for therapeutic development against SARS-CoV-2.

## Results

### Screening of chlorcyclizine-related tricyclic compounds identified LNF as an anti–SARS-CoV-2 compound.

We recently reported that chlorcyclizine and its analog, dichlorcyclizine, which were previously found to have potent antiviral activity against HCV entry, are also effective against SARS-CoV-2 entry (26, 28–30, 33). With this in mind, we tested a large number of related molecules for anti–SARS-CoV-2 activities to identify additional potential candidates for therapeutic development. Structurally and/or functionally related compounds were screened using VSV-pseudotyped virus harboring the S glycoprotein of SARS-CoV-2. SARS-CoV-2 can use both plasma membrane- and endosome-mediated entry pathways, depending on the availability of host proteases (34). To identify compounds with efficacy against both routes of S-mediated entry, all candidate compounds were first screened with Huh7 cells, which are susceptible to endosomal entry. Positive compounds were subsequently screened in 293A2T2 cells, for which SARS-CoV-2 uses TMPRSS2-mediated plasma membrane entry (Supplemental Figure 1; supplemental material available online with this article; https://doi.org/10.1172/jci.insight.182704DS1). Of the 72 compounds initially tested in Huh7 cells, 14 were found to have 50% effective concentration (EC_50_) and 50% cytotoxic concentration (CC_50_) values warranting further testing in 293A2T2 cells. NCGC00346707 (LNF) was the only member of this latter group found to have desirable efficacy and favorable toxicity in 293A2T2 cells (Supplemental Tables 1 and 2). Thus, it was selected for further characterization.

### LNF inhibits SARS-CoV-2 infection in multiple cell lines.

**Figure 1:**
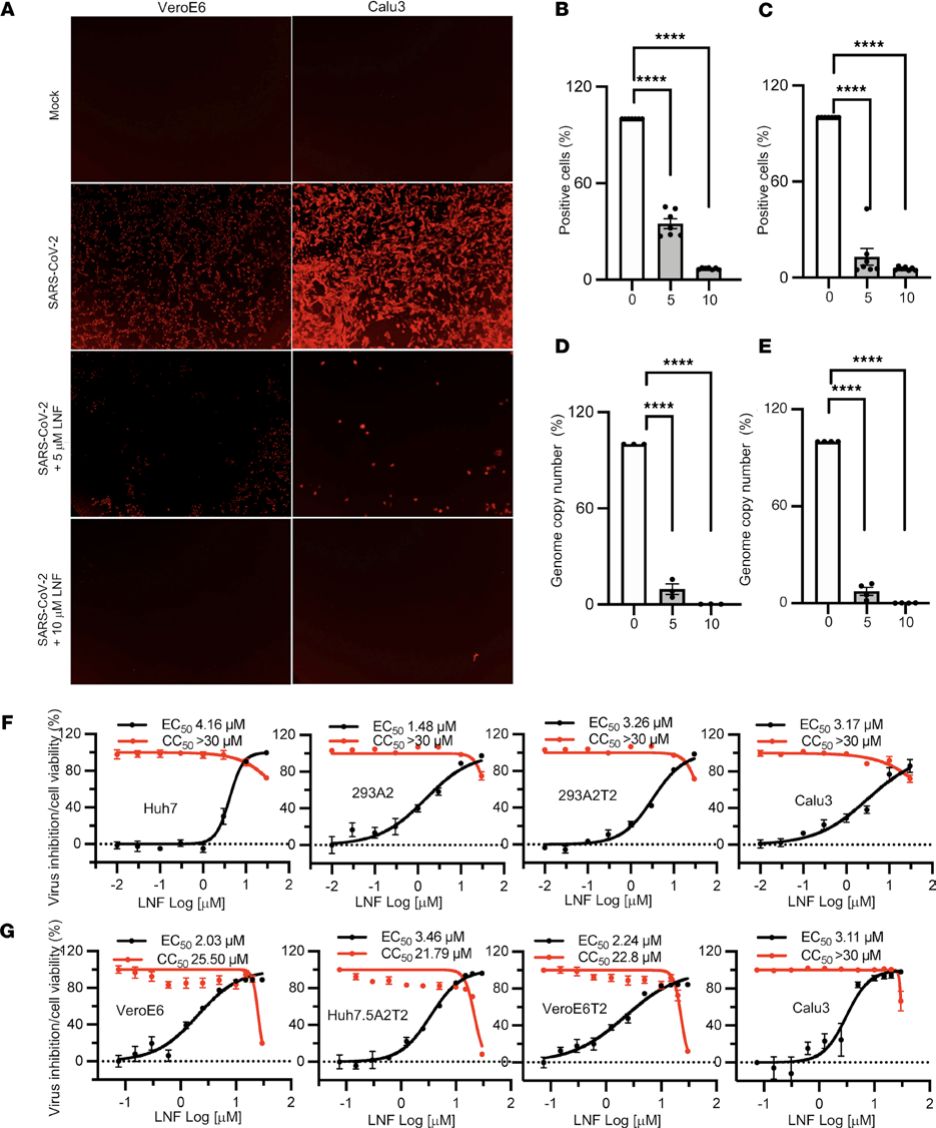
LNF inhibits SARS-CoV-2 infection. (**A**) VeroE6 and Calu3 cells were infected with SARS-CoV-2 and treated with LNF at the time of infection. At 24 hours after infection, cells were fixed and probed with anti-N protein and Alexa Fluor 547–conjugated antibodies. The plates were scanned using an automated plate reader for red fluorescence and images are provided as representative of 28 random areas per treatment group. Original magnification, ×10. (**B** and **C**) The percentage of N-positive cells was determined by counting the number of fluorescent cells followed by the total number of the cells in the same area. Total fluorescence counts were normalized by total number of the cells and percentage positivity was calculated. The results are depicted relative to the DMSO-treated group. The data represent mean ± SEM of 7 replicates and the figure is representative of 3 independent experiments. (**D** and **E**) VeroE6 and Calu3 cells infected with SARS-CoV-2 were treated with 5 and 10 μM LNF. At 48 hours after infection, intracellular RNA was harvested, and genome copy number was determined by qRT-PCR; data represent percentage genome copy number relative to DMSO-treated control. Each data point represents mean ± SEM (*n* = 3) and the figure is representative of 3 independent experiments. ****P < 0.0001 by 1-way ANOVA with Dunnett’s test for multiple comparisons to the control (**B**–**E**). (**F**) Dose-response curve of LNF using VSV-based SARS-SoV-2-S pseudovirus and live infectious SARS-CoV-2-nLUC (**G**). Briefly, the infected cells were treated with multiple concentrations of the drug. At 24 hours after infection, luminescent signals were measured using a POLARstar Omega plate reader. EC_50_ and CC_50_ values were calculated using Prism 7 software. Each data point represents mean ± SEM (*n* = 6). The red and black series represent cell viability and viral inhibition, respectively. The results are representative of 3 independent experiments.

To validate the potential hit LNF, we tested it against infectious SARS-CoV-2 and related viral variants. We infected ACE2- and TMPRSS2-expressing cells with the Wuhan strain. The cells were treated with selected nontoxic concentrations of LNF (5 and 10 μM) and vehicle (DMSO) control. Forty-eight hours after infection, cells were stained for N protein and the relative numbers of N-positive cells were normalized and quantified. We observed that DMSO-treated SARS-CoV-2–infected cells showed strong signals for N protein staining 48 hours after infection (Figure 1A), while the LNF-treated cells showed lower numbers and lower fluorescence signal intensity of N-positive cells. We observed that the extent of viral inhibition was dose dependent (Figure 1, B and C). Similarly, the effect of LNF on the virus-induced cytopathic effect (CPE) was also analyzed. SARS-CoV-2 causes CPE in many of the cell lines and the CPE is often used as a proxy for viral replication. We infected VeroE6 cells with SARS-CoV-2 in the presence of LNF and analyzed the cells’ morphology for CPE. It was noted that LNF treatment rescued the infected cells from virus-induced CPE (Supplemental Figure 2). The CPE-related results further validated our observation that LNF is an anti–SARS-CoV-2 agent. In addition, we examined the direct effect of LNF on viral genome copies in infected cells. A nontoxic concentration (10 μM) of LNF reduced viral genome copy number in infected cells by more than 90% (Figure 1, D and E).

To examine the dose-response characteristics of LNF, we utilized multiple cell lines and virological tools, including a VSV-based (VSV-SARS-CoV-2-S) pseudovirus (33), and an infectious and replication-competent derivative of SARS-CoV-2 that was previously engineered to express a nLUC reporter (35). Dose-response curves and EC_50_ and CC_50_ values for VSV-SARS-CoV-2-S pseudovirus (Figure 1F) and infectious SARS-CoV-2-nLUC (Figure 1G) are shown. EC_50_ values for LNF against VSV-SARS-CoV-2-S pseudovirus ranged from 1.5–4.16 μM and against infectious SARS-CoV-2-nLUC ranged from 2.03–3.46 μM. Thus, LNF inhibits SARS-CoV-2 infection with a high selectivity index in most of the susceptible cells, with a selectivity index (SI = CC_50_/EC_50_) much greater than 10.

### LNF shows a strong synergy with RDV and NRTV and inhibits all major SARS-CoV-2 variants.

**Figure 2:**
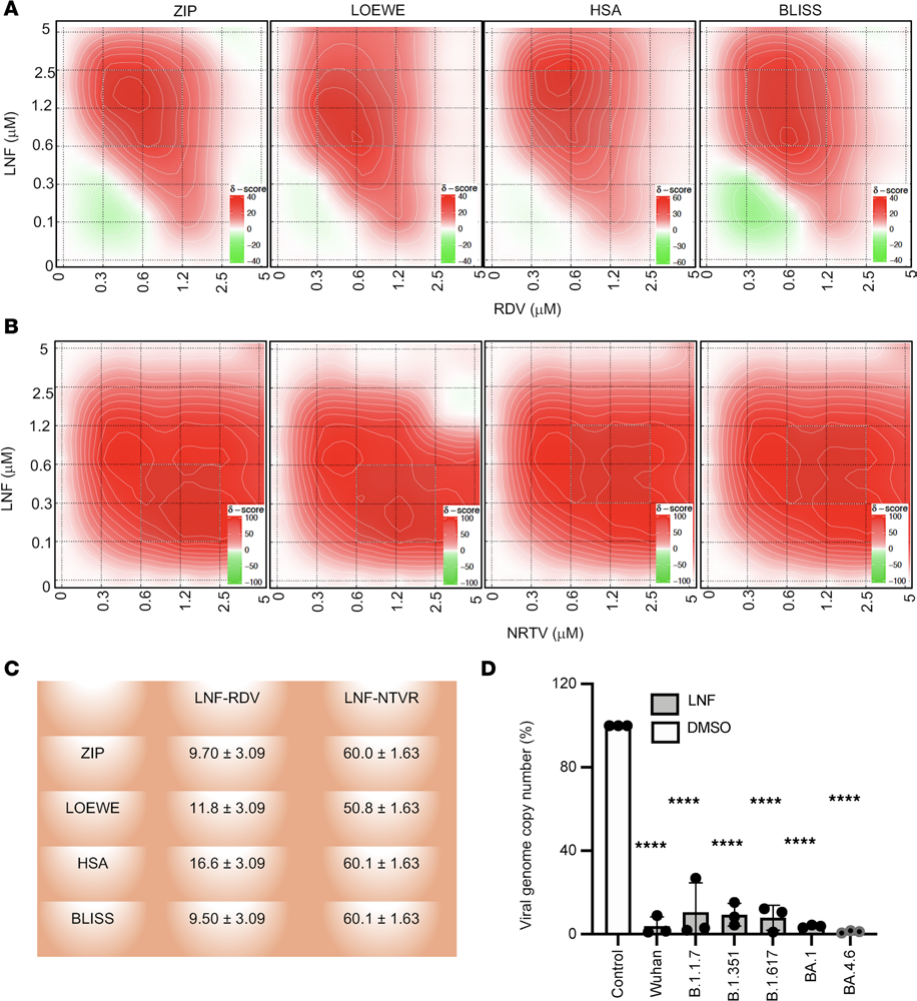
Effect of LNF on SARS-CoV-2 variants and LNF synergy with RDV and NRTV. VeroE6 cells were infected with SARS-CoV-2-nLuc and treated with multiple concentrations of LNF alone and in combination with RDV or NRTV at the time of infection. At 24 hours after infection, the luciferase activity was measured and replication relative to DMSO-treated control was calculated. (**A** and **B**) Inhibition of SARS-CoV-2 replication achieved by a combination of varying concentrations of LNF and RDV (**A**) or NRTV (**B**). Infected cells were treated with compounds at concentrations ranging from 0–5 μM. Viral infectivity was normalized with the untreated (DMSO) infected cells and percentage of inhibition was calculated. Data represent mean values from 3 independent experiments and contour graphs for ZIP, LOEWE, HSA, and BLISS synergy were plotted using Synergyfinder. (**C**) The panel summarizes different synergy score statistics for LNF-RDV and LNF-NRTV combinations. The synergy experiments were repeated 2 times. (**D**) VeroE6 cells were infected with multiple variants of SARS-CoV-2 and cotreated with 10 μM LNF. At 24 hours after infection, total RNA was harvested, and the viral genome copy number was determined by qRT-PCR. The values for the DMSO-treated group were set to 100% and the relative numbers of genome copies were then calculated for the respective LNF-treated groups. The graph values are the mean ± SD of 3 independent experiments. ****P < 0.0001 by 1-way ANOVA with Dunnett’s test for multiple comparisons to the control.

We next tested whether LNF shows any antiviral synergy in combination with other approved anti–SARS-CoV-2 drugs, RDV and NRTV. Antiviral synergy is defined as exhibiting a combined inhibitory effect that is greater than the additive effect of the drugs individually. SARS-CoV-2–infected cells were treated with LNF concentrations ranging from 0–5 μM, alone or in combination with RDV and NRTV. We used SynergyFinder 2 to analyze the synergy of the LNF-RDV and LNF-NRTV combinations (36). When the nLUC activity was measured and analyzed, we observed that LNF showed strong synergy with RDV and NRTV (Figure 2, A and B). The combination of LNF concentration in the range from 1–2.5 μM showed the highest synergy with RDV at concentrations ranging from 0.3–1.0 μM (Figure 2A), while NRTV appeared to be more synergistic with LNF than RDV (Figure 2B). Notably, there are multiple synergy models available, such as highest single agent (HSA), Loewe additivity (LOEWE), Bliss independence (BLISS), and zero interaction potency (ZIP). Hence, we performed statistical analysis of LNF-RDV and LNF-NRTV synergy (36), and calculated ZIP, HSA, BLISS, and LOEWE scores (Figure 2C).

In the VeroE6 cell line, the infection route is predominantly endosomal, and therefore we also performed synergy assays using Calu3 cells, which use the plasma membrane entry pathway. Calu3 cells were treated with a combination of LNF-RDV and LNF-NRTV during infection, and the efficacy was calculated (Supplemental Figure 3, A–C). It was observed that LNF showed a strong synergy with RDV and NRTV in Calu3 cells.

After establishing the anti–SARS-CoV-2 efficacy of LNF in multiple cell lines and its synergistic effect in combination with approved drugs (RDV and NRTV), we then examined its antiviral efficacy against the major variants of SARS-CoV-2 (37). Our results showed that LNF is active not only against the original Wuhan strain of SARS-CoV-2, but also its variants, including the B.1.1.7 (Alpha), B.1.351 (Beta), BA.1.617.2 (Delta), and the BA.1 and BA.4.6 (Omicron) lineages (Figure 2D). We also analyzed LNF-RDV and LNF-NRTV synergy using BA.4.6, a recent variant available in our lab. We infected VeroE6 and treated these cells with multiple combinations of LNF-RDV or LNF-NRTV and showed additive or synergistic effects (Supplemental Figure 3, D and E).

### LNF inhibits SARS-CoV-2 spike protein–mediated cell-cell fusion.

**Figure 3:**
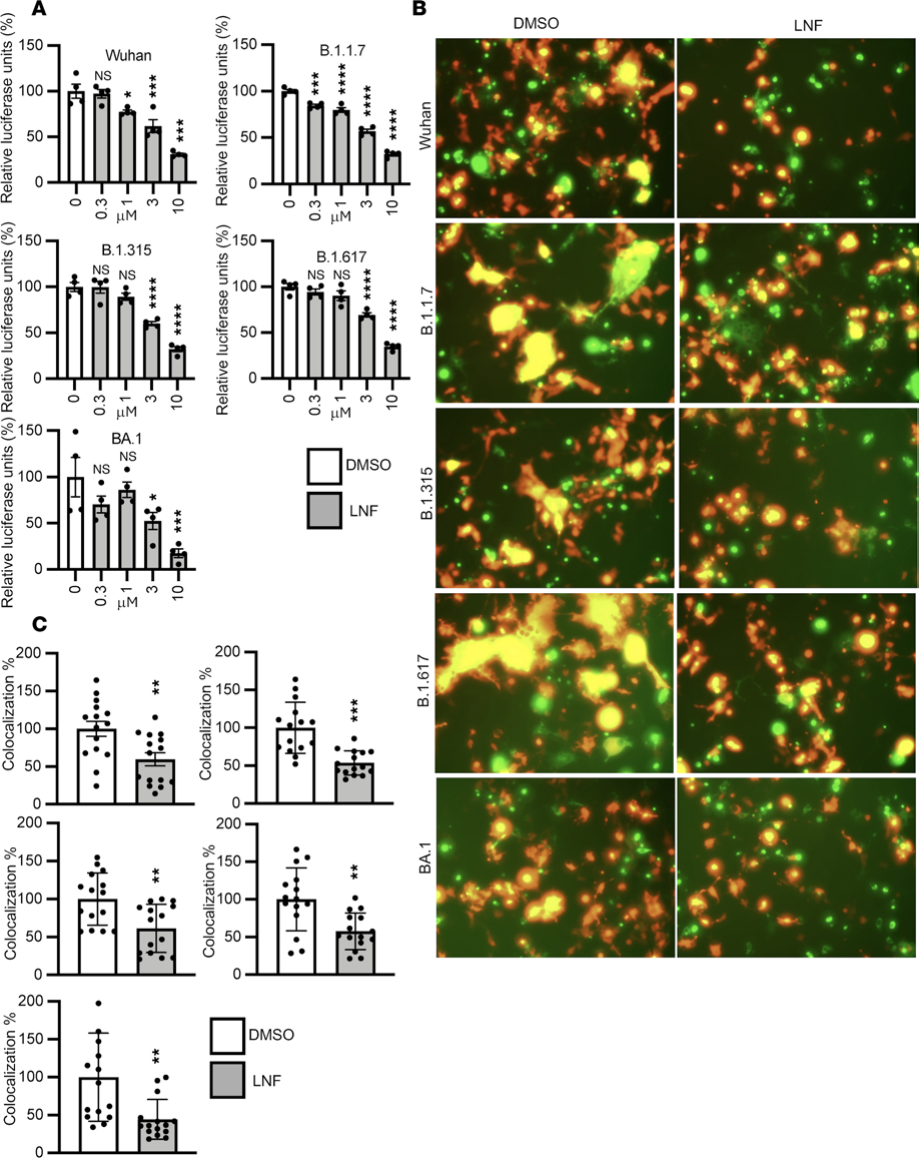
LNF blocks SARS-CoV-2 spike protein–mediated cell-cell fusion. (**A**) Cell-cell fusion assays were performed with LNF. The S-SmBit–transfected donor (HeLa) and the LgBit-transfected recipient (293ACE2) cell mixture was treated with 4 different concentrations of LNF (10, 3, 1, and 0.3 μM) and DMSO as control for 48 hours. After incubation, luminescent signals were measured using a POLARstar Omega plate reader. The values are given as relative luciferase signals and each data point is presented as mean ± SEM (*n* = 4 biological in dependent replicates). NS, *P* > 0.05; *P < 0.05, ***P < 0.001, ****P < 0.0001 by 1-way ANOVA with Dunnett’s test for multiple comparisons to DMSO control. (**B**) LNF (10 μM) was used to treat S-GFP–transfected donor (HeLa) and the RFP-transfected recipient (293ACE2) cell mixture for 48 hours. Representative fields are shown. Original magnification, ×10. (**C**) For quantification, 15 fields were randomly selected from 4 replicates to measure the fused cells under a CellSens fluorescence microscope. ImageJ was used to quantify percentage colocalization signals. White and gray bars represent untreated and treated groups, respectively. **P < 0.01, ***P < 0.0001 by unpaired, 2-tailed *t* test with Welch’s correction. All results are representative of 3 independent experiments.

Previously, we developed 2 binary cell-cell fusion assays: the SmBit-LgBit (split luciferase) and GFP-RFP systems and demonstrated that chlorcyclizine-related compounds inhibited SARS-CoV-2 spike protein–mediated cell-cell fusion (33). Briefly, HeLa cells were used as donor cells and 293ACE2 cells were employed as recipient cells. Since HeLa cells are not susceptible to SARS-CoV-2 infection due to lack of ACE2 expression, they do not undergo self-fusion. HeLa cells were designed to express S-SmBit or GFP fusion protein, while 293ACE2 cells express LgBit or RFP. After successful fusion, luminescent signals and yellow fluorescence signals can be observed based on interaction between SmBit and LgBit and colocalization between GFP and RFP, respectively. To assess whether LNF inhibits Wuhan and other variant S protein–mediated plasma membrane fusion, we tested both SmBit-LgBit and GFP-RFP systems. LNF suppressed cell-cell fusion in a dose-dependent manner for all variants tested in both systems (Figure 3, A and B). In the GFP-RFP system, the colocalization signals representing fused cells (in yellow) were quantified and are shown in Figure 3C.

### Mechanism-of-action studies of LNF in SARS-CoV-2 infection.

**Figure 4:**
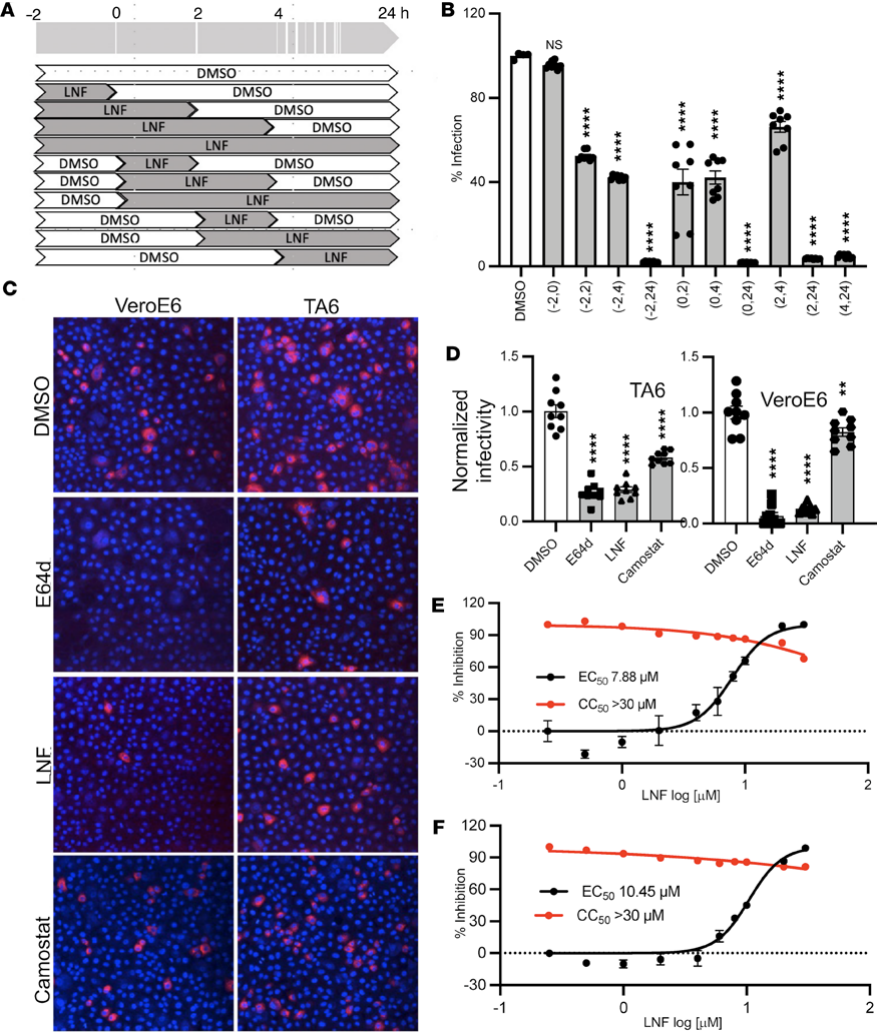
Mechanistic studies of LNF’s antiviral action. (**A**) Schematic of drug treatment plan, where solid dark and empty areas represent the presence and absence of the drug, respectively. The 0 hour represents the time of infection. DMSO was used as control. (**B**) VeroE6 cells were infected with SARS-CoV-2 and treated with DMSO or LNF (10 μM) as described in the Methods and schematic in **A**. The drug was present for the entire duration or removed as per the schematic by replacing with the media containing DMSO only. At 24 hours after infection, the luciferase activity was measured and graphed as percentage replication relative to the untreated, infected control group. Data are presented as mean ± SEM (*n* = 8) and the figure is representative of at least 3 independent experiments. (**C**) Representative microscopic images of VeroTA6 cells (top) and VeroE6 (bottom) that were infected at 0.1 MOI for 4 hours and treated with various compounds (10 μM LNF, 5 μM E64d, and 5 μM camostat). The cells were fixed and stained with antibodies against spike protein (red). Original magnification, ×10. (**D**) The infectivity of virus in the presence of compounds was calculated and normalized to DMSO control. A total of 9 random areas were captured and average infectivity for each treatment group was plotted as mean ± SEM (*n* = 9). This experiment was conducted 2 times. NS, *P* > 0.05; **P < 0.01, ****P < 0.0001 by 1-way ANOVA with Dunnett’s test for multiple comparisons to DMSO control (**B** and **D**). (**E** and **F**) The SARS-CoV-2 replicon and RNA delivery particles (RDPs) were used to prepare the dose-response curve for LNF. For replicon (**E**), Huh7.5 cells were electroporated with the Gluc replicon and treated with multiple concentrations of LNF. After 24 hours, Gluc signal was measured and normalized to vehicle control. The representative graph shows mean values of 3 replicates and error bars indicate SEM (*n* = 4). For RDP assay (**F**), RDPs were generated by *trans* complementation of the SARS-CoV-2 replicon with S protein in producer cells. Huh7.5 ACE-TMPRSS2 cells were then transduced with the Gluc RDPs and treated with multiple concentrations of LNF. Twenty-four hours later, Gluc activity was measured and normalized. The data represent mean values of 3 replicates and error bars indicate SEM (*n* = 4). The results are representative of 3 independent experiments.

We further explored the mechanism of LNF’s antiviral action in SARS-CoV-2 infection. We first performed a time-of-addition assay. The drug was added at various times before and after infection, and the viral replication was measured. We initially tested 3 known compounds, RDV, camostat, and E64d, in our time-of-addition assay (Supplemental Figure 4A). It is well known that RDV inhibits SARS-CoV-2 replication, while E64d and camostat are specific for the entry steps in the viral life cycle. E64d targets the endosomal entry pathway by inhibiting cathepsins, while camostat targets TMPRSS2-mediated membrane fusion. As VeroE6 cells predominantly favor the endosomal route of SARS-CoV-2 infection, we observed that only E64d, and not camostat, was effective in blocking the entry step of the viral life cycle (Supplemental Figure 4, B and C). When E64d was added 2 hours after infection, it showed no inhibitory effect on SARS-CoV-2, indicating viral entry was completed by that time. On the other hand, RDV showed a minimal effect when added for a limited duration at early time points, but showed maximum efficacy when it was added later after infection (Supplemental Figure 4D). Interestingly the time-of-addition assay with LNF suggested more than one mechanism of viral inhibition. When the drug was present during an initial period of viral infection, it showed a modest (50%) but significant effect (Figure 4, A and B). However, the effect was much more pronounced when the drug was present for longer or added at a later time of infection (Figure 4, A and B). We observed a high efficacy of LNF even if the drug was added 4–24 hours after infection. SARS-CoV-2 attachment and entry events are completed 2 hours after infection (Supplemental Figure 4B). Therefore, we reason that LNF likely exerts an inhibitory effect on both viral entry and replication.

To further confirm the effect of LNF on viral entry, we infected cells for only 4 hours in the presence of various inhibitors and then stained for viral spike protein to assess viral entry. In this experiment, we utilized VeroE6 and a modified, more permissive version, the VeroTA6 cell line (VeroE6 with overexpressed human TMPRSS2 and ACE2). In the TA6 cell line after infection (Supplemental Figure 4E), colocalization of the spike protein and LAMP1 signals within vesicle-like structures was detected, suggesting localization in endolysosomes. In the VeroE6 cell line, these signals predominantly colocalized within clustered lysosomal compartments near the nucleus (Supplemental Figure 4E), suggesting somewhat different entry pathway and kinetics between the 2 cells.

To evaluate the entry pathway of the 2 cell lines, we tested the effects of camostat (blocking plasma membrane entry) and E64d (blocking endosomal entry) individually or in combination on SARS-CoV-2 infection (Supplemental Figure 4F). We observed that VeroE6 cells appeared to support only the endosomal route of infection, as only E64d effectively blocked SARS-CoV-2 infection, but not camostat. With the VeroTA6 cell line, neither compound was effective when used individually and only in combination was inhibition evident. These data suggest that VeroTA6 supports both routes of entry and if one of the two routes is blocked, the virus can enter via the other route (Supplemental Figure 4F). Additionally, we examined the impact of LNF on the early stages of viral infection in Calu3, a respiratory epithelium–derived cell line that is more biologically relevant for SARS-CoV-2 infection. Since viral entry in these cells primarily occurs through plasma membrane fusion, this experiment helped to determine whether the observed effect in the Vero cell lines within the 0–2 hour period is associated with the inhibition of endocytosis. We observed that LNF had little or no effect on the early events of viral infection in Calu3 cells. (Supplemental Figure 4G). These data support the idea that the modest impact of LNF during the initial stage of viral infection in other cell lines is related to endocytosis.

Next, we evaluated camostat, E64d, and LNF in inhibiting viral entry using the above immunofluorescence entry assay. As expected, E64d, but not camostat, exhibited a robust inhibitory effect in VeroE6. Like E64d, LNF inhibited viral entry, suggesting that part of its antiviral effect derives predominantly from targeting the endosomal pathway of entry (Figure 4, C and D). Lysosomal acidification plays a major role in the endosomal pathway of viral infection. We therefore evaluated the effect of LNF on the cell’s lysosomal compartment. We stained the control and LNF-treated cells with LysoTracker dye and visualized the cells for fluorescence. Interestingly, LNF-treated cells exhibited significantly higher fluorescence intensity after staining with LysoTracker (Supplemental Figure 5A). Chloroquine (CQ) and E64d were added as control drugs. As expected, CQ-treated cells showed a significant reduction in fluorescence intensity, while E64d that inhibits cathepsins showed no effect (Supplemental Figure 5A). We next tested the effect of LNF on a lysosomal enzyme, cathepsin L, a member of endosome/lysosome-associated enzymes that are important for SARS-CoV-2 entry by cleaving the S2′ site on the S protein. We treated the cells with multiple concentrations of LNF and measured cathepsin L activity. We observed no effect of LNF on cathepsin activity at any concentration used (Supplemental Figure 5B). Thus, LNF probably targets and enhances lysosomal activity to degrade incoming SARS-CoV-2.

### Effect of LNF on SARS-CoV-2 replication.

As shown above, LNF appears to have a potent antiviral effect after viral entry. To further study this observation, we used SARS-CoV-2 replicon and replicon delivery particle (RDP) methods (38). The replicon system bypasses the initial attachment and entry events and represents only viral replication. We showed that LNF was active against the replicon, with an EC_50_ of 7.8 μM (Figure 4E). LNF was similarly effective in the RDP system, with an EC_50_ of 10.4 μM (Figure 4F).

Interestingly, LNF has been predicted by in silico modeling to interact with NSP12 and NSP7 (part of the viral polymerase complex) of SARS-CoV-2 and possibly inhibits viral replication (39). We thus tested whether LNF has a direct inhibitory effect on the viral RdRp activity using an in vitro assay with purified components (40). In this experiment, the polymerase activity, as shown by primer extension, was inhibited by the positive control (compound TEMPOL) but not affected by LNF, suggesting that LNF is not a direct inhibitor of RdRP (Supplemental Figure 6).

### Inhibition of farnesyl transferase mediates the antiviral effect of LNF.

**Figure 5:**
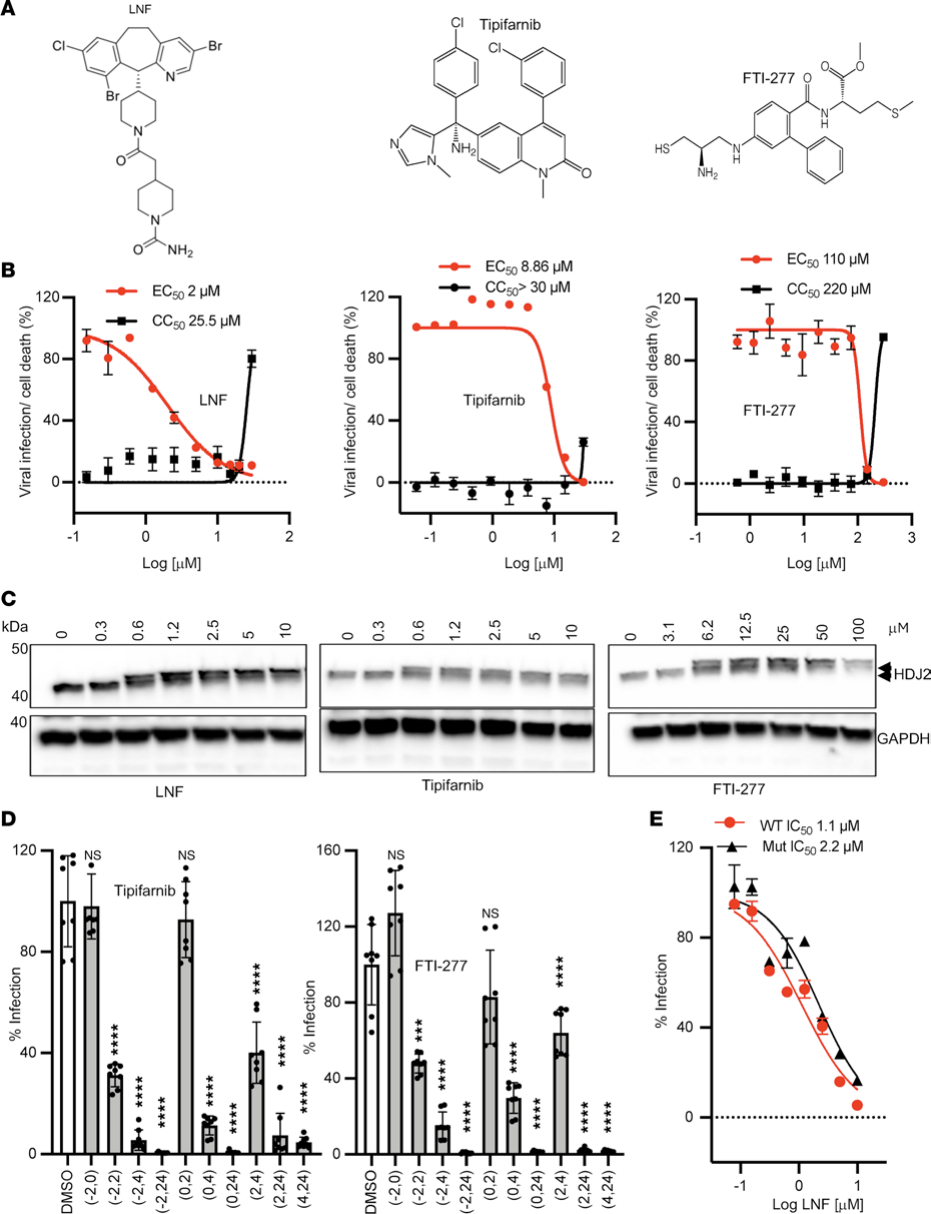
Effect of other FTase inhibitors on SARS-CoV-2 infection. (**A**) The chemical structures of LNF, tipifarnib, and FTI-277. (**B**) Dose-response curves of LNF, tipifarnib, and FTI-277 were prepared and relative replication was graphed. The VeroE6 cells were infected with SARS-CoV-2-nLuc and treated with these 3 drugs followed by luciferase activity measurement at 24 hours after infection. The red and black series represent percentage viral luciferase and cell viability, respectively. All data points represent mean ± SEM (*n* = 4) and the figure is representative of 3 independent experiments. The red and black series represent the level of viral infection and cell death, respectively. (**C**) Shift in the mobility of HDJ2 protein was assessed using Western blotting. The cells were treated with multiple concentrations of the drug and at 24 hours after treatment, the lysates were prepared and resolved using SDS-PAGE followed by transfer of the separated proteins to a nitrocellulose membrane. The membrane was probed with anti-HDJ2 (Invitrogen) and anti-GAPDH (Santa Cruz Biotechnology). A shift in electrophoretic mobility of HDJ2 is indicated by arrows. This experiment was conducted 2 times, and the blots are representative. (**D**) Time-of-addition assay was performed using VeroE6 cells treated with tipifarnib (10 μM) and FTI-277 (300 μM). Please see the schematic in Figure 4A. The infected cells were treated with the drug for varying durations of pre- and postinfection times and the luciferase activity was measured. The relative replication was plotted, where all data points represent mean ± SEM (*n* = 8) and the figure is representative of 3 independent experiments. NS, *P* > 0.05; ***P < 0.001, ****P < 0.0001 by 1-way ANOVA with Dunnett’s test for multiple comparisons to DMSO control. (**E**) Efficacy of LNF was tested in VeroE6 cells transfected with WT and mutant FNTB plasmids. At 48 hours after transfections, cells were infected with SARS-CoV-2-nLuc and luciferase activity was measured at 24 hours after infection. Data are presented as mean ± SEM (*n* = 4). The results are representative of 3 independent experiments.

The outstanding question regarding the mechanism of action of LNF is whether farnesyl transferase (FTase) enzyme inhibition is responsible for LNF’s effect against SARS-CoV-2 and not a result of an off-target effect. If this were the case, we reasoned that other FTase inhibitors would also show efficacy against SARS-CoV-2. We tested 2 additional, well-known FTase inhibitors, tipifarnib and FTI-277, which are structurally distinct from LNF (Figure 5A). Tipifarnib inhibited SARS-CoV-2 infection with a comparable EC_50_/CC_50_ dose response (Figure 5, A and B). FTI-277 showed efficacy against SARS-CoV-2 infection, with an EC_50_ higher than those of the other 2 FTase inhibitors (Figure 5B).

We next examined the effects of FTase-specific inhibition by the 3 inhibitors on HDJ2, a cellular protein. HDJ2 is a direct substrate of FTase and its farnesylated (lower band) and unfarnesylated (upper band) forms can be easily differentiated by electrophoretic mobility (41) (Figure 5C). Using this assay, we observed that the effective inhibitory doses of the 3 compounds correlated well with their anti–SARS-CoV-2 activities (Figure 5C). The result also explains why FTI-277 has a lower potency in inhibiting SARS-CoV-2 (higher EC_50_) because of its weaker anti-FTase activity, supporting the notion that the anti–SARS-CoV-2 activity associated with LNF is likely mediated by its inhibitory effect on cellular FTase.

In the time-of-addition assay, the efficacy of LNF was predominantly observed to be targeting the late stage of viral replication. However, LNF did show modest efficacy in targeting initial steps of the viral life cycle. Thus, LNF targets both entry and replication stages of the SARS-CoV-2 life cycle. We performed the time-of-addition experiment with tipifarnib and FTI-277 to determine whether farnesylation inhibition is responsible for both effects. Both tipifarnib (Figure 5D) and FTI-277 (Figure 5D) showed a similar pattern of efficacy. Like LNF, they showed a modest effect on the early stage of infection, while the efficacy was much higher in the late stage of the viral life cycle.

FTase and geranylgeranyl transferase (GGTase) are 2 major cellular enzymes that catalyze protein prenylation. To determine whether geranylgeranylation is also involved here, we treated SARS-CoV-2–infected cells with GGTI2418, a known specific inhibitor of GGTase (42). We observed that the GGTase inhibitor had no effect on viral replication (Supplemental Figure 7A). To further validate that the function of FTase mediates the antiviral effect of LNF in SARS-CoV-2 infection, we employed a genetic knockdown strategy. We reasoned that FTase knockdown should mimic the effect of LNF and show reduced SARS-CoV-2 infection. Using siRNA against the *FNTB* gene, we observed approximately 80% knockdown (Supplemental Figure 7B), but no effect on SARS-CoV-2 infection (Supplemental Figure 7C). Notably, despite significant knockdown, the remaining FTase was still capable of farnesylating cellular proteins efficiently, as shown by the HDJ2 shift assay (Supplemental Figure 7B). We next tried to knock out the *FNTB* gene using CRISPR/Cas technology. We were not able to generate cell clones with homozygous knockout, probably reflecting the essential role of the *FNTB* gene in cells.

The RAS family of proteins are known to be farnesylated by FTase for proper signaling and have been implicated in viral infections (42, 43). We reasoned that if RAS were involved here, then si*RAS* knockdown should reduce viral replication like LNF. We first used VSV-SARS-CoV-2-S pseudovirus and assayed its replication in NRAS-, HRAS-, and KRAS-depleted cells. Despite effective depletion of target gene expression by respective siRNAs, we observed no reduction in SARS-CoV-2-S pseudovirus replication (Supplemental Figure 8, A and B). We also analyzed the role of RAS proteins in SARS-CoV-2 infection. Similarly, we did not see any significant reduction in viral infectivity in RAS-depleted cells (Supplemental Figure 8, C and D). These results suggest that only FTase, and not GGTase, is important for viral replication, and that the effects of LNF are likely not mediated by RAS signaling.

An LNF-resistant mutant of FTase with a specific mutation (W106R) in the active site has previously been identified (44). LNF’s efficacy against SARS-CoV-2 was analyzed in cells overexpressing either wild-type (WT) or W106R mutant (MT) forms of FTase. We observed that LNF was nearly 2-fold less effective in cells expressing the mutant form of FTase, although the difference was not statistically significant (Figure 5E). This nonsignificant reduction could be explained by the presence of endogenous WT FTase in these cells that may reduce the effect of the transfected mutant FTase. However, the trend is supportive of the role of FTase in mediating the antiviral effect of LNF.

### LNF treatment showed reduced viral titer and improved tissue pathology in SARS-CoV-2–infected mice.

Before conducting the efficacy experiments using the K18-*hACE2* mouse model (45), we performed a pharmacokinetics experiment in this mouse strain and harvested various tissues for determination of LNF concentration after a single dose (40 mg/kg [MPK]) of LNF via intraperitoneal administration. The LNF pharmacokinetics results are summarized in Supplemental Table 3A. LNF distributed widely to various mouse tissues except the brain. The lung-to-plasma AUC ratio was approximately 3, suggesting a preferential lung accumulation. The lung concentration of LNF (8.17 μM) at 24 hours was higher than its in vitro EC_50_ (1–4 μM) at 24 hours. We decided to use 40 MPK twice daily in the in vivo efficacy experiment. Fifty MPK twice daily dosing has been tested in preclinical mouse studies without any toxicity.

**Figure 6:**
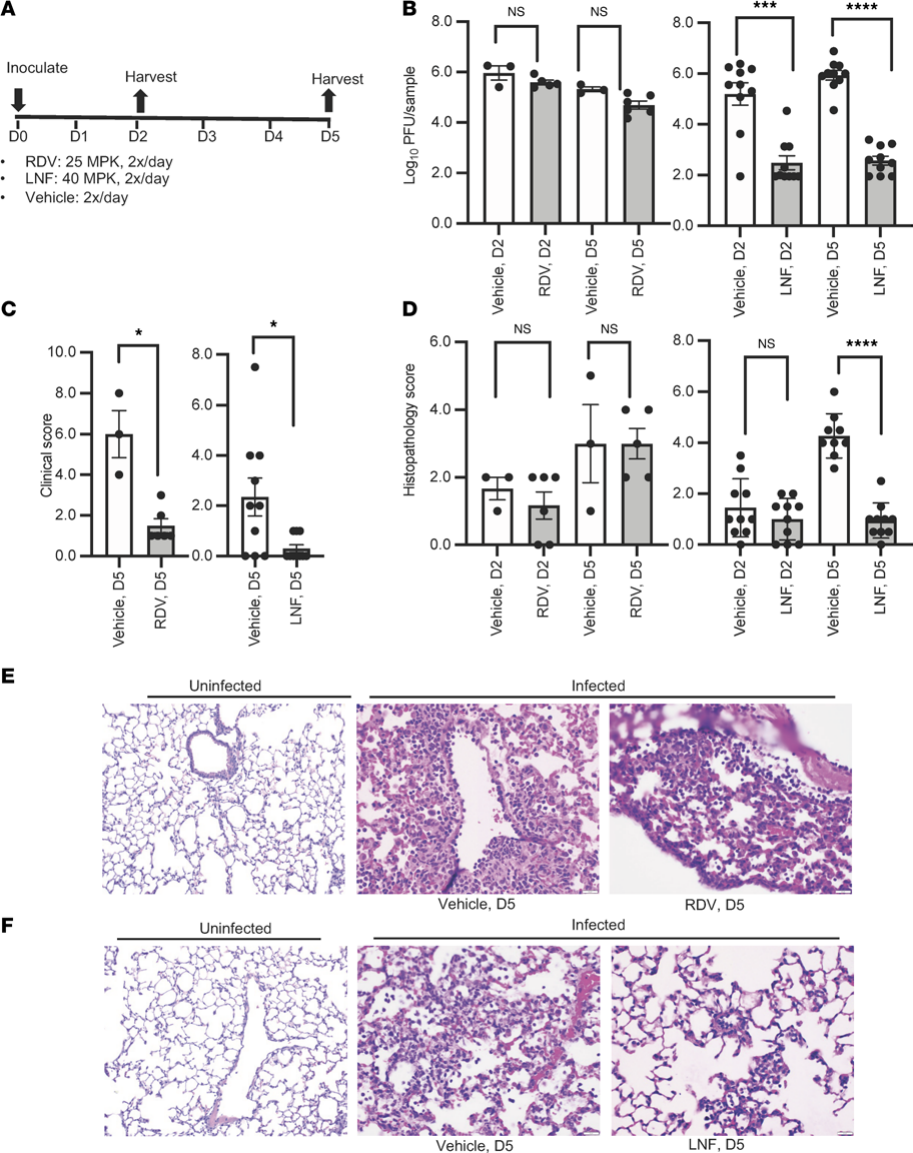
Efficacy of LNF in an animal model. (**A**) Drug treatment scheme showing how the K18-*hACE2* mice were infected with SARS-CoV-2 and treated intraperitoneally with drugs. (**B**) Tissues harvested on days 2 and 5 (D2 and D5) after infection were analyzed for viral titer as described in the Methods. (**C**) Composite clinical scores calculated based on 4 disease parameters related to posture, behavior, and activity, breathing, and weight loss each rated from 0 to 3 (maximum total score 12). All results are representative of 3 independent experiments. (**D**) Tissue sections were individually graded from 0–3 based on degree of alveolar inflammation as well as degree and frequency of necrosis/hyaline membrane formation and perivascular inflammation. These were then summed for a composite histopathology score. All graphs show mean values ± SEM. NS, *P* > 0.05; *P < 0.05, ***P < 0.0001, ****P < 0.0001 by unpaired, 2-tailed *t* test with Welch’s correction (**B**–**D**). (**E**) Representative H&E-stained histopathology images of lung from uninfected (left image) and infected mice treated with vehicle (middle image) or RDV (right image) sacrificed on day 5. Vehicle- and RDV-treated mice exhibited similar lesions on day 5. Lesions were characterized by moderate to large numbers of predominantly lymphocytes, with some histiocytic cells and rare neutrophils centered on vessels (middle image). Low to moderate numbers of similar infiltrates with slightly more neutrophils were often present in alveoli (right image). (**F**) Representative H&E-stained histopathology images of lung from uninfected (left image) and infected mice treated with vehicle (middle image) or LNF (right image) sacrificed on day 5. Vehicle-treated mice exhibited similar lesions, which were characterized by neutrophils and fewer lymphocytes and histiocytic cells present within alveoli and surrounding vessels (middle image). In contrast, LNF-treated mice had no to low amounts of inflammation within alveoli and surrounding vessels (right image). Scale bars: 20 μm (**E** and **F**).

K18-*hACE2* mice were infected with SARS-CoV-2 and treated intraperitoneally with LNF or RDV (and vehicle control for each study), as shown in Figure 6A. LNF treatment significantly lowered the viral titer in the lung. On days 2 and 5 after infection, the viral titers were nearly 2-log lower than the vehicle-treated group, whereas the RDV-treated mice did not show much reduction in viral titers (Figure 6B). The composite clinical score of infected animals was calculated and both LNF- and RDV-treated animals exhibited much improved disease parameters (Figure 6C). Lung tissues obtained from LNF-, RDV-, and vehicle-treated groups were examined for pathology. The degree of alveolar inflammation and degree and frequency of necrosis/hyaline membrane formation and perivascular inflammation were analyzed and graded from 0 to 3. The LNF-treated group on day 5 showed reduced inflammation, which is reflected in terms of significantly lower histopathology score, compared with the vehicle-treated mice (Figure 6D). The RDV-treated group, however, showed similar histological scores to those of the vehicle-treated mice on day 5.

Lung histopathology revealed lesions that were characterized by moderate to large numbers of predominantly lymphocytes with some histiocytic cells and rare neutrophils centered on vessels in vehicle-treated mice (Figure 6E). In RDV-treated animals, low to moderate numbers of similar infiltrates with slightly more neutrophils were often present in alveoli (Figure 6E). In contrast, LNF-treated mice had no to low levels of inflammation within alveoli and surrounding vessels (Figure 6F), compared with the vehicle-treated mice that exhibited tissue lesions characterized by neutrophils, lymphocytes, and histiocytic cells present within alveoli and surrounding vessels (Figure 6F).

Since LNF is used as an oral drug, we thought to test the efficacy of orally administered LNF. First we performed a single-dose pharmacokinetic experiment with 25 MPK via oral gavage. The data indicated lower tissue concentrations and shorter half-lives of LNF as compared with the intraperitoneal dosing (Supplemental Table 3). Because of solubility issues with LNF, we proceeded with 50 MPK twice daily dosing for this experiment. The mice were infected and treated with LNF as depicted in Supplemental Figure 9A. On day 2, LNF-treated animals showed significantly lower viral titers in the lung (Supplemental Figure 9B). When lung sections were analyzed for the presence of alveolar inflammation, and degree and frequency of necrosis/hyaline membrane formation and perivascular inflammation, the LNF-treated group also showed a significantly lower histopathology score, compared with the vehicle-treated mice (Supplemental Figure 9C). In the vehicle group on day 2, minimal perivascular inflammation composed of mainly lymphocytes, plasma cells, and macrophages was noted (arrows in Supplemental Figure 9D). Moreover, occasional thickening of alveolar septal interstitium by similar infiltrates (arrowheads) was detected. The LNF group on day 2 also exhibited minimal perivascular inflammations (arrows) that were not different from those of the vehicle group (Supplemental Figure 9D). However, on day 5, the vehicle group showed medium to high numbers of lymphocytes, plasma cells, and macrophages cuffing vessels (arrows). Many samples exhibited expansion of the alveolar interstitium by lymphocytes, macrophages, and plasma cells (arrowheads). There were frequently low to medium numbers of neutrophils and macrophages within alveolar spaces. However, the LNF group on day 5 showed minimal perivascular inflammation composed of mainly lymphocytes, plasma cells, and macrophages (arrows). Mild increases in neutrophils and macrophages within the alveolar space were also seen (arrowheads) (Supplemental Figure 9D). In this experiment, the overall antiviral effect of LNF appeared to be less than that of the intraperitoneal experiment, which is not unexpected because of the less favorable pharmacokinetic parameters associated with oral dosing.

## Discussion

The COVID-19 pandemic has entered its fourth year and continues to exact a heavy public health threat worldwide with a recent resurgence of infections and hospitalizations (46–48). While successful development of preventive vaccines has substantially lessened the viral transmission and public health burden, effective therapies are necessary to reduce disease severity, mortality, and long-term consequences. As vaccine efficacy may wane against emerging variants, antiviral development will continue to play an important role in controlling this pandemic as well as any future emerging viral pathogens. Current approved treatments, when used within a short period of initial infection, are effective but suboptimal (8, 13, 24).

In this study, we identified and demonstrated that LNF, at clinically relevant doses, is an effective antiviral against SARS-CoV-2 and its variants in cell culture. It also acts synergistically with 2 approved antivirals (RDV and Paxlovid). In the K18-*hACE2* mouse model, LNF improved lung pathology and suppressed pulmonary viral levels. LNF was also more potent than RDV, a clinically approved drug against SARS-CoV-2. LNF appears to target multiple steps of SARS-CoV-2 infection, including viral entry and replication, with the latter being the predominant mode of action. During viral entry, LNF inhibits the virus-cell membrane fusion process based on cell-cell fusion assays, similar to what we have shown previously for other compounds (33). At this point, whether LNF acts directly on the viral fusion mechanism or indirectly via a host-mediated pathway is not clear. LNF, by blocking cell-cell fusion and syncytia formation that is a pathological hallmark of COVID-19 disease (49, 50), may also reduce pathology associated with SARS-CoV-2 infection. LNF appears to act at the endosomal step of viral entry, possibly by enhancing lysosomal activities to degrade incoming virus based on the imaging studies (Figure 4, C and D, and Supplemental Figure 5). On the other hand, LNF potently inhibited viral replication in a cell-based replicon system, but did not directly target viral RdRp in a cell-free replicase assay (Figure 4, E and F, and Supplemental Figure 6). The time-of-addition experiments are also consistent with the multistep antiviral activity of LNF.

LNF is a potent inhibitor of the cellular enzyme FTase, which consists of 2 subunits, α (FNTA) and β (FNTB), with FNTB containing the enzyme active site (51). FTase catalyzes farnesylation of numerous cellular proteins (52). LNF was first developed for cancer therapy because the RAS family of proteins, which are farnesylated and frequently activated in many cancers (51). It was subsequently approved by the FDA to treat Hutchinson-Gilford progeria syndrome (HGPS), in which the mutant form of the progerin protein is farnesylated and causes progeria. Blocking progerin’s farnesylation by LNF is effective in reducing disease progression in HGPS (52, 53). The clinically approved dose for HGPS is up to 150 mg/m^3^ body surface area (in adults, ~150 mg) twice daily, which is comparable to the equivalent dose (40 MPK, twice daily) used for our efficacy study in hK18-ACE2 mice (54, 55).

Protein prenylation, in which a protein is enzymatically modified either by incorporation of farnesyl group (catalyzed by FTase) or geranylgeranyl isoprenoid (catalyzed by GGTase), is a posttranslational modification that is functionally important for many proteins (56). Our mechanistic studies demonstrated that the main antiviral effect of LNF is mediated via FTase inhibition. First, a GGTase inhibitor showed no effect against SARS-CoV-2. Second, structurally unrelated inhibitors of FTase exerted similar antiviral effects that are consistent with their dose-response pharmacological properties. Third, LNF-resistant mutation confers a reduced efficacy of LNF in anti–SARS-CoV-2 activity. An extensive sequence search of all encoded proteins of SARS-CoV-2 did not reveal a canonical farnesylation motif (CAAX, where C = cysteine, A = aliphatic amino acid, and X = any amino acid). Thus, the antiviral target of LNF is likely a farnesylated cellular protein.

LNF has been shown to inhibit hepatitis D virus (HDV) replication and is currently being tested in clinical trials as a treatment for HDV (57). In this case, it is well known that the HDV large δ antigen, which is essential for HDV assembly, contains a CAAX motif that is farnesylated by FTase (58, 59). During the preparation and review of this paper, Weber et al. reported the efficacy of LNF against SARS-CoV-2. However, their study did not address any mechanistic aspects and mainly provided efficacy data in cell culture (60).

More than 100 cellular proteins have been shown or predicted to be farnesylated by FTase and farnesylation is essential for their functions (56). The RAS family of proteins are well-known targets of FTase and previous studies have suggested a role of these proteins in various viral infections (56, 58, 61). Our experiment in which we knocked down various *RAS* genes by siRNA did not show any notable effect on the antiviral activity of LNF. A recent study suggested that a zinc finger antiviral protein (ZAP), which is farnesylated and can be induced by interferons, is a potent antiviral gene against SARS-CoV-2 (62). LNF, by blocking the farnesylation of this antiviral protein, should exert a proviral effect on SARS-CoV-2 replication, which is opposite to the observed antiviral effect described here. Thus, we reason that LNF inhibits the farnesylation of a yet unknown host protein that is essential for viral replication. Regarding inhibition of viral entry by LNF, our data also support the idea that FTase inhibition is involved, although we cannot rule out that LNF may have a direct effect on viral entry. Further studies are thus necessary to identify the responsible gene(s) for the antiviral effect(s) of LNF. Since FTase modifies many cellular proteins and thereby regulates diverse pathways, LNF may have an effect against other viruses as well. A recent study also demonstrated the antiviral effect of LNF against respiratory syncytial virus (63).

Recent approaches using in silico modeling and molecular simulation identified LNF as a potential hit that may target the SARS-CoV-2 life cycle (64). Ruan et al. predicted that LNF can bind to the active pockets between NSP12 and NSP7 of SARS-CoV and SARS-CoV-2, and therefore may inhibit SARS-CoV-2 replication (39). All these predictions were based on modeling approaches and need experimental validation. Our studies of LNF’s anti–SARS-CoV-2 activity did not point to these predicted targets.

Based on our extensive in vitro and in vivo experiments, we showed that LNF, at clinically relevant doses, is an effective antiviral against SARS-CoV-2 infection. LNF has been tested and used extensively in both adult and pediatric populations with excellent long-term safety profile. Thus, our results suggest that LNF is a promising antiviral against SARS-CoV-2 worthy of further clinical assessment for treatment of COVID-19 as a repurposing drug.

## Methods

Further information, including reagent and antibody details, can be found in Supplemental Methods.

*Sex as a biological variable*. Our study examined male and female animals, and similar findings are reported for both sexes.

*In vivo SARS-CoV-2 challenge and treatment*. All animal experiments were carried out in Animal Biosafety Level 3 (ABSL3) facilities at the Infectious Disease Unit (IDU) at Virginia Tech in accordance with national and institutional guidelines. K18-*hACE2* (Tg) C57BL/6J mice of both sexes (Jackson Laboratory) were anesthetized and challenged by intranasal inoculation of 1 × 10^5^ PFU of SARS-CoV-2 strain USA-WA1/2020 in 50 mL PBS. Animals were treated twice daily with either 25 MPK RDV subcutaneously, 40 MPK LNF intraperitoneally, or with vehicle polyethylene glycol 300, 20% 2-hydroxypropyl-β-cyclodextrin (w/v), and ethanol (5:4:1, v/v) only intraperitoneally twice daily. Mice were also observed and assessed for morbidity of disease at each treatment point, with scoring based on percentage weight loss from starting weight, body condition, respiration, and general activity. On days 3 and 5 after infection, mice were euthanized via CO_2_ inhalation. Following perfusion with sterile 1× PBS, lungs were collected and fixed by inflation and immersion in buffered 10% formalin. Lung slices were subjected to H&E staining for histopathologic examination. Sections of lung were scored according to the following parameters: airway changes including epithelial necrosis, luminal inflammation, and peri-airway inflammation; alveolar changes including necrosis, fibrin, air space inflammation, and septal inflammation; and perivascular inflammation.

For oral dosing studies, K18-*hACE2* C57BL/6J mice were anesthetized using 3.5% isoflurane and infected with 1 × 10^5^ PFU SARS-CoV-2-WA diluted in sterile 1× PBS. Animals were treated twice daily with 50 MPK LNF via oral gavage. These animals were monitored for clinical disease for 5 days. At indicated time points, mice were euthanized with CO_2_, whole blood was collected by cardiac puncture, and serum was isolated and stored at –80°C. Lungs were removed and lobes collected for subsequent analysis. The left lung was inflated with formalin and fixed for histopathology assessments and the cranial lobe was homogenized and evaluated for viral titer. For histopathologic evaluation, lungs were fixed by immersion in buffered formalin, embedded in paraffin, and stained with H&E for analysis. Lung sections were scored based on assessments of mononuclear and polymorphonuclear cell infiltration, perivascular and peribronchiolar cuffing, estimates of the percentage of lung involved with disease, and epithelial cell defects based on the severity/extent of damage to the cell barrier as previously described (65, 66). Reviewers were blinded to genotype and treatment.

*Virus, cells, and infection*. All the viral stocks were produced, maintained, and handled in an appropriate biosafety level laboratory and as per the SOPs formulated by the NIH. All the variants of SARS-CoV-2 were obtained from the SARS-CoV-2 core facility (SVC) at the National Institute of Allergy and Infectious Diseases (NIAID), NIH, and BEI Resources (https://www.beiresources.org/). The references for all the variants are SVG-001/USA-WA1 (Wuhan); SVG-015 UK/CA B.1.1.7; SVG-019 RSA 1.351 501Y; SVG-028 Delta; SVG-053 Omicron SARS-CoV-2/human/USA/HI-CDC-4359259-001/2021; SARS-CoV-2, HCoV-19/USA/MD/HP35538/2022 (BA.4.6). All these strains were propagated in VeroE6 cells expressing TMPRSS2. The viral isolates were sequence confirmed and titrated using a plaque assay. The aliquots of viral stocks were kept in a –80°C freezer for future use. Once the aliquot was taken out to use, the remaining amount was discarded and never re-frozen. The method to produce recombinant VSV-SARS-CoV-2-S-GFP virus and its use for initial screening has been described previously (33). The SARS-CoV-2 replicon and RDPs were produced and used as described previously (38).

VeroE6 (ATCC), VeroE6-TMPRSS2 (obtained from the SVC, NIAID), and Huh7-TMPRSS2 (gift from Charles Rice’s lab, Rockefeller University, New York, New York, USA) were maintained in DMEM plus 10% FBS. Calu3 (ATCC) and Caco2 (ATCC) were maintained in DMEM plus 20% FBS. For infection, cell monolayers were infected with virus at 0.1 MOI and incubated at 37°C for 2 hours with gentle shaking every 15 minutes. Following attachment, the virus was removed, the cells were washed with PBS, and fresh media were added. The infected cells were then incubated for and processed for downstream steps as per the need of the experiments.

*Plasmid construction*. Codon-optimized SARS-CoV-2 S cDNA plasmid was purchased from Genscript. The C-terminus of the SARS-CoV-2 S protein (containing an ER retention signal) was truncated by 20 amino acids to enhance virus yield (67, 68). A single nucleotide mutation was introduced at nucleotide 3759 (C to A) for SARS-CoV-2 using an In-Fusion cloning kit (Takara) according to the manufacturer’s instructions, which resulted in an amino acid change from Cys to a stop codon. In brief, pCMV-VSV-G (Addgene plasmid number 8454) (69) was digested with BamHI to remove the VSV-G sequence. The S sequence was then assembled into the CMV promoter-containing backbone. The Alpha (69/70 deletion, N501Y, D614G, and P681H) (70), Beta (K417N, E484K, N501Y, and D614G) (71), and Delta (T19R, G142D, 156/157 deletion, R158G, L452R, T478K, D614G, P681R, and D950N) (24) variant S constructs were generated using a Q5 Site-Directed Mutagenesis Kit (New England BioLabs). Omicron (A67V, Δ69-70, T95I, G142D, Δ143-145, Δ211, L212I, Ins214EPE, G339D, S371L, S373P, S375F, K417N, N440K, G446S, S477N, T478K, E484A, Q493R, G496S, Q498R, N501Y, Y505H, T547K, D614G, H655Y, N679K, P681H, N764K, D796Y, N856K, Q954H, N969K, and L981F) variant S construct was synthesized by a commercial source (Genscript). The assembled constructs were used for VSV pseudotyped virus generation.

*Statistics*. In all figures, the data are represented as mean ± SD or mean ± SEM, which is clearly mentioned in the respective figure legends. The tests for evaluating the significance were appropriately applied and a *P* value of less than 0.05 was considered significant.

*Study approval*. All in vitro and animal experiments were conducted in accordance with the policies set forth by the NIH.

*Data availability*. Values for all data points in graphs are reported in the Supporting Data Values file. New analytic code was not generated during this study.

### Author contribution

MK, PI, and TJL designed the research studies. MK, PI, SBP, HMI, IRL, ML, AG, ESJ, SCO, QZ, NM, JKJ, BL, WH, AQW, XX, ZH, and WZ conducted experiments. MK, PI, SBP, HMI, IRL, ML, AG, ESJ, SCO, QZ, NM, JKJ, BL, WH, AQW, XX, ZH, and WZ acquired data. MK, PI, HMI, IRL, SCO, QZ, WH, AQW, XX, ZH, WZ, YY, TR, ICA, CMR, and TJL analyzed data. IRL, TR, CMR, ICA, YY, and TJL provided reagents. MK and TJL wrote the manuscript.

## Supplementary Material

Supplemental data

Unedited blot and gel images

Supporting data values

## Version 1 12/03/2024

In-Press Preview

## Version 2 01/09/2025

Electronic publication
