# Supplementary material for: Repurposing of lonafarnib as a treatment for SARS-CoV-2 infection
Source: JCI Insight. 2025 Jan 9;10(1):e182704. doi: 10.1172/jci.insight.182704 (PMC11721293; doi:10.1172/jci.insight.182704)
Supplement: Supplemental data [file jciinsight-10-182704-s103.pdf]

Repurposing of lonafarnib as a treatment for SARS-CoV-2 infection

Supplementary Information

Chemicals and antibodies

Remdesivir, Lonafarnib, FTI-277 and GGTase inhibitor were purchased from MedKoo Biosciences Morrisville NC, USA. E64d (MilliporeSigma), camostat (MilliporeSigma) and tipifarnib (MedChem Express) were also used. All the stocks were prepared in DMSO.

The antibodies anti-GAPDH (Santa Cruz Biotechnology), anti-HDJ2 (Invitrogen, PA5-81929), anti-FNTB (Abcam, ab236649), anti-actin (Cell Signaling, 4967), anti-Spike S (GenTex, GTX632604), anti-LAMP1 (Cell Signaling, 9091) , Goat anti-Rabbit Alexa 488 (Thermo Fisher, A32731) , Goat anti-Mouse Alexa 568 (Thermo Fisher, A11004) and anti-N protein (GeneTex, GTX135357) were used as per manufacturer's instructions.

Immunofluorescence

SARS-CoV-2 infected cells were fixed with 4% paraformaldehyde (ChemCruz, Dallas, TX) at 48 h post-infection. The cells were then treated with 0.5% Triton X-100 in PBS (phosphate-buffered saline). The permeabilized cells were blocked by 5% bovine serum albumin (BSA, MilliporeSigma) in PBS. Anti-N protein (GeneTex), and Alexa Fluor 555 donkey anti-Mouse IgG (Thermo Fisher Scientific, A31570) were used for primary and secondary immunolabelling respectively. The plates were scanned in Spectramax Cell Imaging System (Invitrogen) for red fluorescence. The red signal counts were then normalized with total cell count in each well and the percent positivity was determined for each well.

For immunofluorescence experiments related to entry inhibition, cells were infected with SARS-CoV-2, treated with various inhibitors, and then stained with antibodies. First cells were fixed in

PBS containing 4% paraformaldehyde for 15 min at room temperature and washed three times with PBS followed by permeabilization with PBS containing 0.2% saponin and 10% fetal bovine serum. Cells were then stained with primary antibody overnight at 4 °C followed by secondary antibodies (1:2000). The dilution factors for primary antibodies are: Mouse anti-Spike of SARS-CoV-2, 1:500; Rabbit anti-Lamp1 1:1000. Fluorescence confocal images were acquired by a Nikon CSU-W1 SoRa microscope equipped with a temperature control enclosure and a CO_2 control at NIDDK imaging core. 3D image reconstructions and analyses were done by Imaris software (Licensed to NIH). Fluorescence intensity was analyzed by open-source Fiji software. To this end, images were converted to individual channels, and regions of interest were drawn for measurement. Statistical analyses were performed using GraphPad Prism 9.0. *P* values were calculated by one-way ANOVA by GraphPad Prism 9.0. Images were prepared by Photoshop and Illustrator (Adobe). Data processing and reporting are adherent to the community standards.

Luciferase and viability assay

For drug toxicity assays**,** we used PhosphoWorks™ Luminometric ATP Assay Kit (AAT Bioquest). White 96-well plates (Greiner Bio-One) with seeded density of 10000 cells per well were used. Cells were treated with various dilutions of the drugs and at 24 h post treatment, luciferase activity was measured using POLARstar Omega plate reader (BMG LABTECH).

Tissue processing and viral titer determination

After collection, lungs were flash-frozen on dry ice, and stored at -80°C for a minimum of 48 h. After freezing lung tissues were homogenized at 30osc/sec for 5 minutes in 0.5mL of viral diluent (RPMI with 2% FBS, 10mM HEPES) using 2.8mm stainless steel beads. Viral titer was determined by plaque assay on these lung homogenates.

Cell fusion assay

For the cell-cell fusion assay, additional sequences were inserted to the above SARS-CoV-2 truncated S construct to generate truncated S-SmBit and truncated S-GFP constructs. The same BamHI-digested pCMV-VSV-G backbone was used for both constructs. For S-SmBit construction, the truncated S sequence and P2A-SmBit sequence were amplified by PCR and assembled with the digested backbone using the In-Fusion cloning kit according to manufacturer's instruction. For S-GFP construct generation, the truncated S and P2A sequence from the above construct and GFP were amplified by PCR and assembled as above. RFP and LgBit expressing constructs were generated similarly.

Quantitative RT-PCR

The purification of total RNA was done by Trizol Method. The relative levels of RNA were analyzed by quantitative real time reverse transcriptase-polymerase chain reaction (qRT-PCR) using the Verso 1-step RT-qPCR Kit (Thermo Fisher Scientific) by using following heating/annealing/extension conditions: 1 cycle of 50°C for 2 min, 60°C for 30 min, and 95°C for 15 min, followed by 50 cycles of PCR at 95°C for 20 s, and 62°C for 1 min. *GAPDH* gene primers (F 5'- GGAGCGAGATCCCTCCAAAAT-3' and R 5'- GGCTGTTGTCATACTTCTCATGG-3') were used for normalization and SARS-CoV-2 *N3* gene primers (F 5'-ATGCTGCAATCGTGCTACAA -3' and R 5'-GACTGCCGCCTCTGCTC-3') were used for viral RNA detection.

Cell-cell fusion assay

To assess SARS-CoV-2 S protein-mediated cell-cell fusion, the plasmid expressing GFP fused to the C-terminally truncated SARS-CoV-2 S or plasmid expressing SmBit from the NanoBit system (Promega) fused to S were transfected in HeLa cells (donor cells). In recipient cells (HEK293ACE2), plasmid expressing RFP or LgBit, were transfected. The fusion events between donor cells with GFP and recipient cells with RFP were visualized and quantified. In the fused cells, SmBit and LgBit proteins' proximity results in luminescent signals that allow the quantification of fusion events between SmBit expressing donor cells and LgBit expressing recipient cells. In brief, first the cells were transfected with the appropriate plasmids described above. At 24 h post-transfection, 5 mM EDTA and Accutase was used to dissociate the cells. Then, 30,000 cells per well of donor cells and 15,000 cells per well of the recipient cells were seeded in 96 well plates (clear bottom black plate for fluorescence measurement, white plate for luciferase measurement) and co-cultured with inhibitor compounds in DMEM for 48 h. From 4 replicates, 20 random microscopic field were selected and imaged under CellSens fluorescence microscope (Olympus, Tokyo, Japan) and colocalization signal was quantified using ImageJ (National Institutes of Health, Bethesda, MD, USA). For SmBit-LgBit system, Furimazine (substrate for Nanoluciferas) was added and Nanoluciferase activity was measured by POLARstar Omega plate reader (BMG LABTECH).

***In vitro* mRNA synthesis**

Wild-type and mutant (W106R) *FNTB* mRNAs were synthesized *in vitro*. The *FNTB* mRNA sequence used in this study is available from the National Center for Biotechnology Information (Reference Sequence: NM_002028.4). Briefly, Addgene plasmid 70379 (a gift from Dominic

Esposito) was taken and the *FNTB* mRNA sequence was cloned into an expression vector with a T7 promoter upstream and a 3X glycine linker with a Flag tag downstream of the *FNTB* mRNA sequence. The HiScribe T7 ARCA kit (New England Biolabs) was employed to synthesize mRNAs, resulting in capped analogs and tails. The reactions were carried out following the manufacturer's instructions. Subsequently, the synthesized mRNAs were purified using the phenol/chloroform extraction and ethanol precipitation method. The transfection of *FNTB* mRNAs was conducted using the TransIT-mRNA Transfection kit (Mirus). Initially, mRNAs were diluted in Opti-MEM (Gibco). Subsequently, Boost reagent and TransIT-mRNA were added sequentially. After an incubation period of 2 to 5 minutes at room temperature, the RNA-lipid complexes were transferred to the corresponding wells of the pre-seeded plates for transfection.

Pharmacokinetics study

K18-*hACE2*(Tg) C57Bl/6J mice were obtained and were maintained at the NIH animal facilities where all protocols were followed by the Division of Veterinary Resources and the Animal Care and Use Committee at the NIH. Single-dose LNF at 40 mg per kg (MPK) was administered IP or 50 MPK for oral dosing by oral gavage. LNF was formulated in polyethylene glycol 300, 20% 2-hydroxypropyl-b-cyclodextrin (w/v) and ethanol (5:4:1, v/v). At various time points (0.083, 0.25, 0.5, 1, 2, 4, 7 and 24 h) after inoculation of mice (n=3 per time point), blood/plasma, brain, liver, kidney, heart and lung were harvested, immediately frozen and stored at -80°C before analysis, and then subjected for determination of LNF concentration using standard ultraperformance liquid chromatography-mass spectrometry (UPLC-MS/MS). The pharmacokinetic (PK) parameters were calculated using the non-compartmental approach (Model 200) of Phoenix WinNonlin, version 8.1 (Certara, St. Louis, MO). The area under the plasma concentration versus time curve (AUC) was

calculated using the linear trapezoidal method. The slope of the apparent terminal phase was estimated by log linear regression using at least 3 data points (except brain tissue) and the terminal elimination rate constant (k) was derived from the slope. $AUC_{0-¥}$ was estimated as the sum of the AUC_{0-t} (where t is the time of the last measurable concentration) and Ct/k. The apparent terminal half-life ($t_{½}$) was calculated as 0.693/k.

***In vitro* RdRp Activity Assay (RNA Extension Assay)**

All RNA oligonucleotides were purchased from Dharmacon (Horizon Discovery). RNA primers were radiolabeled at the 5' end with [$\gamma-$ ^{32}P] ATP (Perkin Elmer) using the T4 polynucleotide kinase from the KinaseMax 5' End-Labeling Kit (Thermo Fisher Scientific). The pair of primer/template oligonucleotides consisted of the 13-mer primer 5'-rArGrGrUrArArUrArArArArUrU-3' and the 29-mer template 5'-rUrUrUrUrArArUrC rCrUrArArArCrGrArArArUrUrUrUrArUrUrArCrCrU-3'. The primer/template annealing, and extension assay were performed as per previous report. Three concentrations of LNF (0.1, 1 and 10 μM) were tested. TEMPOL 100 μM and DMSO were used positive and negative control respectively.

Antiviral testing

Antiviral testing was performed via the NIAID's Antiviral Program for Pandemics (APP). We utilized its pre-clinical services program and obtained in vitro antiviral testing against multiple viruses.

Supplementary Table 1. Results of initial screening of the compounds for antiviral efficacy and cell toxicity.

Compound Identification	Huh7			HEK293A2T2			Compound Property
	EC_{50} (μM)	CC_{50} (μM)	Max %	EC_{50} (μM)	CC_{50} (μM)	Max %	
**NCGC00016949**	>30	>30	29.7	ND	ND	ND	AH
**NCGC00018255**	>100	>30	26.3	ND	ND	ND	AH
**NCGC00018271**	2.2	>30	96.1	>30	>30	22.2	SR
**NCGC00346845**	22.5	24.6	64.2	ND	ND	ND	SR
**NCGC00346851**	14.9	>30	68.3	ND	ND	ND	SR
**NCGC00347049**	>100	>30	ND	ND	ND	ND	SR
**NCGC00351281**	>100	>30	21.1	ND	ND	ND	SR
**NCGC00351673**	28.1	>30	77.7	ND	ND	ND	SR
**NCGC00016421**	25.2	>30	58.1	ND	ND	ND	SR
**NCGC00351675**	>100	>30	17.1	ND	ND	ND	SR
**NCGC00354586**	>100	>30	ND	ND	ND	ND	SR
**NCGC00179384**	5.5	ND	77.8	>30	>30	31.1	SR
**NCGC00343775**	3.0	11.2	77.3	>30	>30	ND	SR
**NCGC00343774**	11.1	14.4	66.4	>30	>30	ND	SR
**NCGC00345880**	5.1	13.4	99.6	12.4	>30	75.2	SR
**NCGC00351414**	4.1	>30	83.6	>30	>30	ND	SR
**NCGC00245963**	2.6	>30	99.5	>30	>30	ND	SR
**NCGC00351413**	2.6	12.9	67.9	ND	ND	ND	SR
**NCGC00354584**	2.0	15.6	100.0	ND	ND	ND	SR
**NCGC00347039**	4.0	14.1	70.8	ND	ND	ND	SR
**NCGC00347044**	3.6	ND	90.9	ND	ND	ND	SR
**NCGC00015246**	>30	22.0	65.8	ND	ND	ND	SR
**NCGC00345879**	3.0	28.0	100.0	ND	ND	ND	SR
**NCGC00164603**	>30	ND	ND	ND	ND	ND	AH
**NCGC00165791**	>100	ND	ND	ND	ND	ND	AH
**NCGC00177979**	4.3	ND	96.4	ND	ND	ND	AH
**NCGC00015817**	4.5	ND	87.0	ND	ND	ND	AH
**NCGC00015273**	>30	ND	ND	ND	ND	ND	AH
**NCGC00015195**	3.1	ND	100.0	ND	ND	ND	AH
**NCGC00016395**	5.2	ND	51.1	ND	ND	ND	AH
**NCGC00015243**	>30	ND	ND	ND	ND	ND	AH
**NCGC00015619**	>30	ND	ND	ND	ND	ND	AH
**NCGC00181101**	>30	ND	ND	ND	ND	ND	AH
**NCGC00015453**	>30	ND	ND	ND	ND	ND	AH

**NCGC00016710**	2.9	>30	96.6	>30	>30	40.9	AH
**NCGC00018296**	>30	ND	54.8	ND	ND	ND	AH
**NCGC00014679**	1.5	ND	100.0	ND	ND	ND	AH
**NCGC00018271**	2.2	ND	96.1	ND	ND	ND	AH
**NCGC00167434**	>30	ND	24.2	ND	ND	ND	AH
**NCGC00014485**	2.8	ND	90.9	ND	ND	ND	AH
**NCGC00015704**	>30	ND	ND	ND	ND	ND	AH
**NCGC00015771**	>30	ND	25.1	ND	ND	ND	AH
**NCGC00183837**	>30	ND	ND	ND	ND	ND	AH
**NCGC00016624**	12.8	ND	57.9	ND	ND	ND	AH
**NCGC00159325**	1.5	>30	98.9	>30	>30	46.0	AH
**NCGC00183046**	1.1	26.3	96.3	>30	>30	46.1	AH
**NCGC00182053**	>30	ND	ND	ND	ND	ND	AH
**NCGC00016672**	11.1	ND	77.6	ND	ND	ND	AH
**NCGC00182073**	>30	ND	ND	ND	ND	ND	AH
**NCGC00015227**	>30	ND	ND	ND	ND	ND	AH
**NCGC00182075**	>30	ND	ND	ND	ND	ND	AH
**NCGC00016913**	8.4	ND	79.3	ND	ND	ND	AH
**NCGC00015822**	>30	ND	49.2	ND	ND	ND	AH
**NCGC00015580**	>30	ND	ND	ND	ND	ND	AH
**NCGC00014482**	2.5	>30	94.3	>30	>30	30.0	SR
**NCGC00016613**	>30	ND	ND	ND	ND	ND	AH
**NCGC00024714**	>30	ND	ND	ND	ND	ND	AH
**NCGC00160492**	>30	ND	ND	ND	ND	ND	AH
**NCGC00015252**	12.3	ND	52.7	ND	ND	ND	AH
**NCGC00016939**	>30	ND	37.9	ND	ND	ND	AH
**NCGC00182705**	>30	ND	45.5	ND	ND	ND	AH
**NCGC00167472**	13.6	ND	59.8	ND	ND	ND	AH
**NCGC00015335**	17.9	ND	17.9	ND	ND	ND	AH
**NCGC00183847**	11.4	>30	64.0	18.4	>30	54.6	AH
**NCGC00162100**	23.3	ND	59.8	ND	ND	ND	AH
**NCGC00015829**	1.5	18.4	100.0	>30	>30	37.0	AH
**NCGC00181013**	>30	ND	ND	ND	ND	ND	AH
**NCGC00263532**	>30	ND	ND	ND	ND	ND	AH
**NCGC00166141**	26.3	ND	35.5	ND	ND	ND	AH
**NCGC00178145**	>30	ND	ND	ND	ND	ND	AH
**NCGC00485995**	17.9	ND	ND	ND	ND	ND	AH
**NCGC00346707**	4.2	>30	100.0	3.3	>30	100.0	SR

ND- Not determined.

AH-Antihistamine.
SR- Structurally related.
Blue color compounds were positive in Huh7 cells and not active in HEK293A2T2 cells; the red color compound was active in both, Huh7 and HEK293A2T2 cells.
Not highlighted compounds are those that showed no activity in Huh7 and therefore not tested in HEK293A2T2 cells.

Supplementary Table 2. Chemical structure of each compound used in screening.

NCGC00016949<br>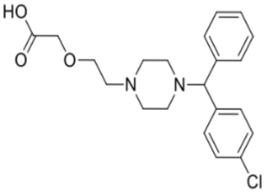	NCGC00018255<br>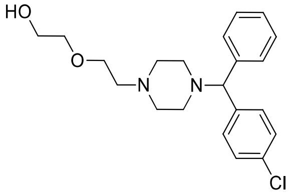	NCGC00018271<br>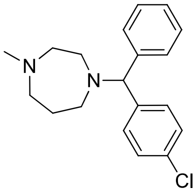	NCGC00346845<br>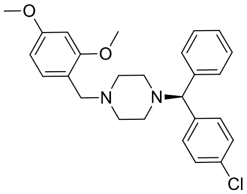
NCGC00346851<br>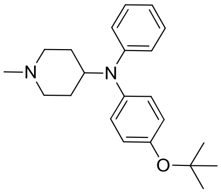	NCGC00347049<br>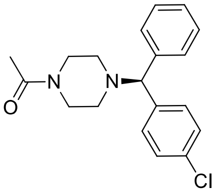	NCGC00351281<br>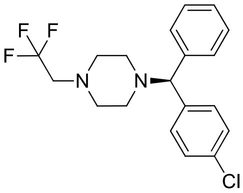	NCGC00351673<br>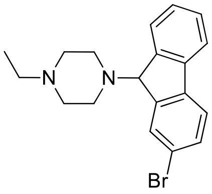
NCGC00016421<br>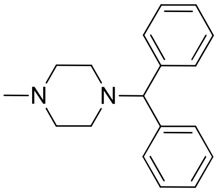	NCGC00351675<br>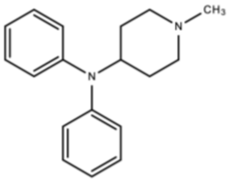	NCGC00354586<br>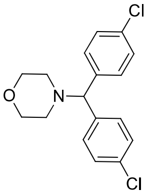	NCGC00179384<br>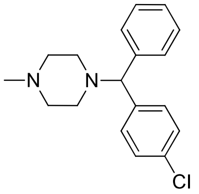
NCGC00343775<br>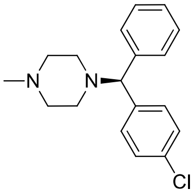	NCGC00343774<br>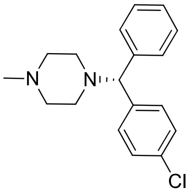	NCGC00345880<br>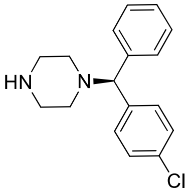	NCGC00351414<br>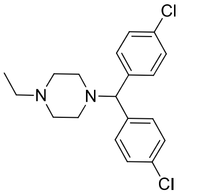
NCGC00245963<br>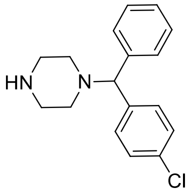	NCGC00351413<br>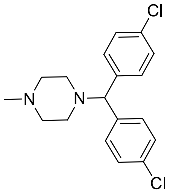	NCGC00354584<br>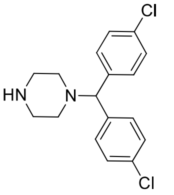	NCGC00347039<br>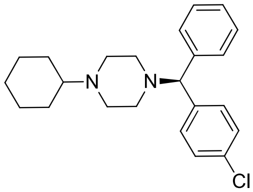
NCGC00347044<br>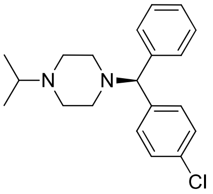	NCGC00015246<br>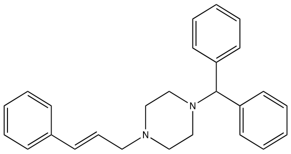	NCGC00345879<br>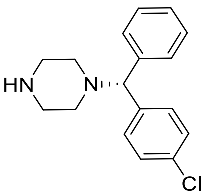	NCGC00164603<br>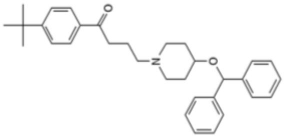

NCGC00165791<br>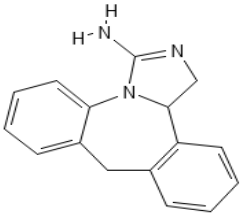	NCGC00177979<br>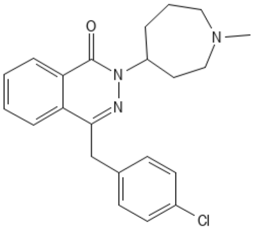	NCGC00015817<br>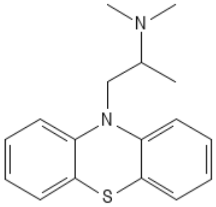	NCGC00015273<br>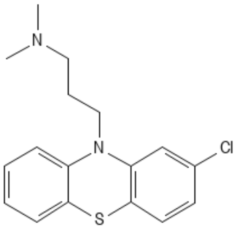
NCGC00015195<br>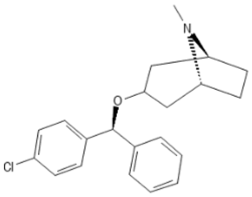	NCGC00016395<br>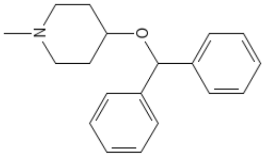	NCGC00015243<br>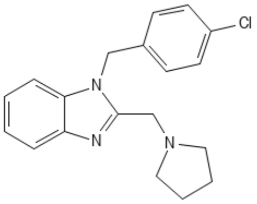	NCGC00015619<br>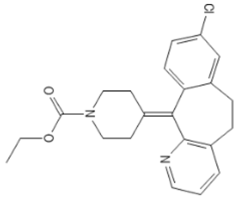
NCGC00181101<br>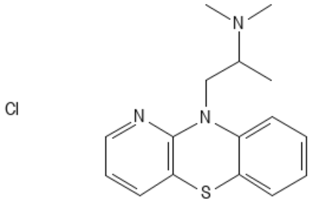	NCGC00015453<br>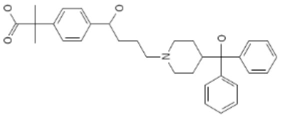	NCGC00016710<br>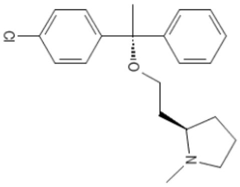	NCGC00018296<br>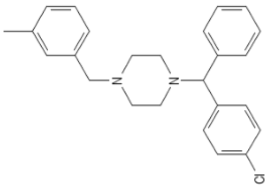
NCGC00014679<br>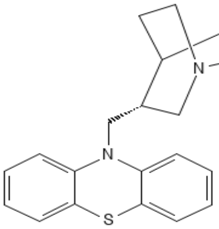	NCGC00018271<br>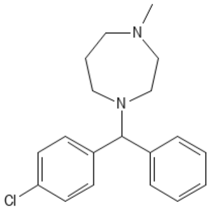	NCGC00167434<br>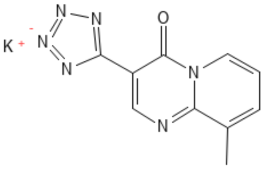	NCGC0001448501<br>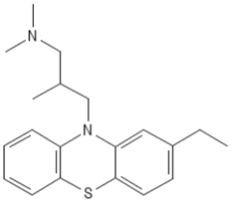
NCGC00015704<br>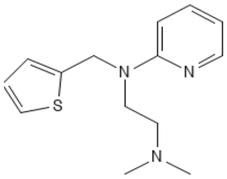	NCGC00015771<br>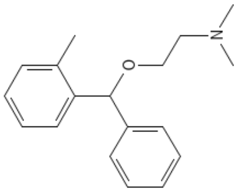	NCGC00183837<br>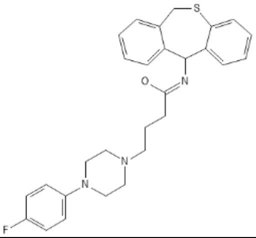	NCGC00016624<br>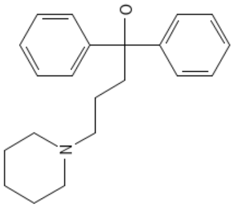
NCGC00159325<br>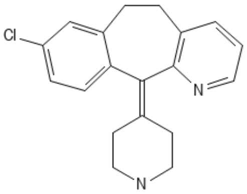	NCGC00183046<br>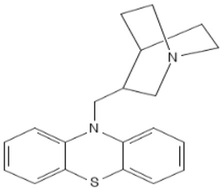	NCGC00182053<br>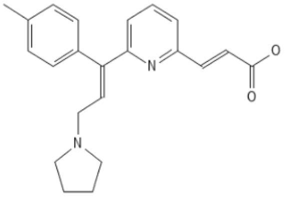	NCGC00016672<br>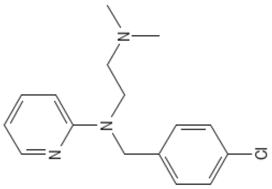

NCGC00182073<br>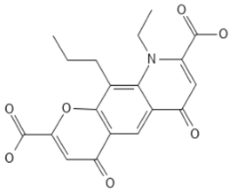	NCGC00015227<br>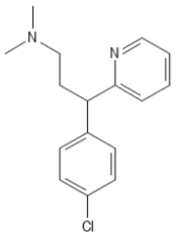	NCGC00182075<br>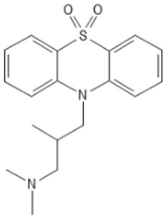	NCGC00016913<br>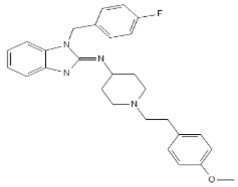
NCGC00015822<br>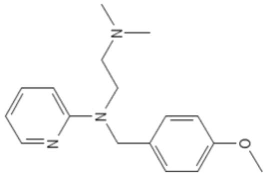	NCGC00015580<br>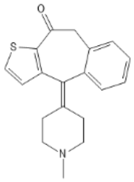	NCGC00014482<br>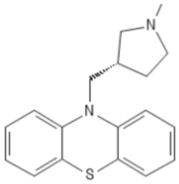	NCGC00016613<br>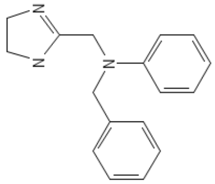
NCGC00024714<br>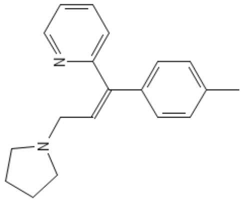	NCGC00160492<br>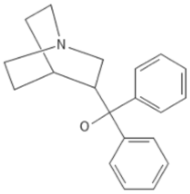	NCGC00015252<br>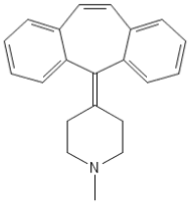	NCGC00016939<br>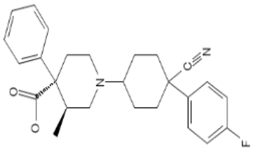
NCGC00182705<br>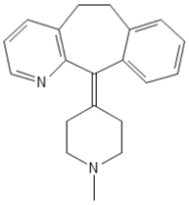	NCGC00167472<br>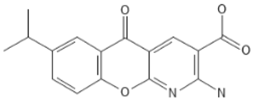	NCGC00015335<br>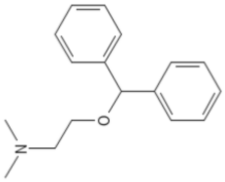	NCGC00183847<br>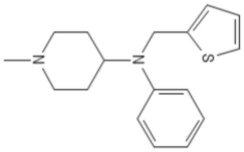
NCGC00162100<br>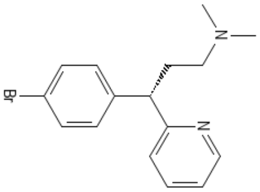	NCGC00015829<br>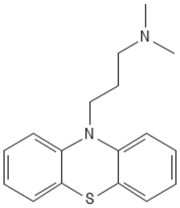	NCGC00181013<br>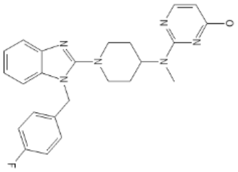	NCGC00263532<br>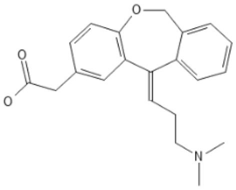
NCGC00166141<br>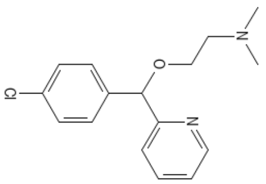	NCGC00178145<br>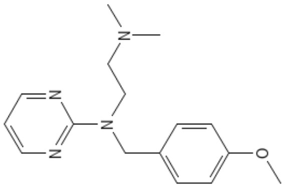	NCGC00485995<br>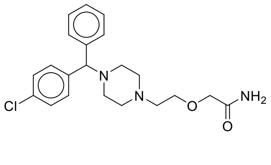	NCGC00346707<br>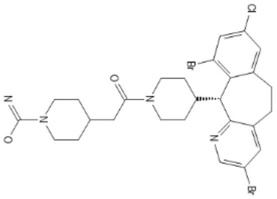

Supplementary Table 3A. Pharmacokinetics of lonafarnib in mouse plasma, brain, liver, kidney, heart, and lung after single IP administration.

	LNF 40 mg/ml					
**Sample**	Plasma	Brain	Liver	Kidney	Heart	Lung
**$AUC_{0\rightarrow inf.}$ (μM*h)**	253	29	3063	699	987	788
**$t_{1/2}$ (h)**	8.8	8.3	10.7	9.0	10.1	8.7
**T_{max} (h)**	0.08	7	0.25	1	0.5	0.25
**C_{max} (μM)**	63.3	1.46	224	46.0	94.1	93.5
**C_{24h} (μM)**	2.76	0.36	40.3	8.24	11.4	8.17
**AUC ratio (Tissue/PL)**		0.12	12	2.8	3.9	3.1

Supplementary Table 3B. Pharmacokinetics of lonafarnib in mouse plasma, brain, liver, kidney, heart, and lung after PO administration.

	LNF 25 mg/ml					
**Sample**	Plasma	Brain	Liver	Kidney	Heart	Lung
**$AUC_{0\rightarrow inf.}$ (μM*h)**	53	3	279	113	97	129
**$t_{1/2}$ (h)**	1.4	2.4	1.7	2.4	2.2	2.1
**T_{max} (h)**	7	7	7	1	1	0.25
**C_{max} (μM)**	3.8	0.21	20	9.3	8.6	33
**AUC ratio (Tissue/PL)**		0.056	5.2	2.1	1.8	2.4

$AUC_{0\rightarrow inf.}$: area under the curve from zero to infinity; $t_{1/2}$: half-life; T_{max}: time to reach the maximal concentration; C_{max}: maximal concentration after administration.

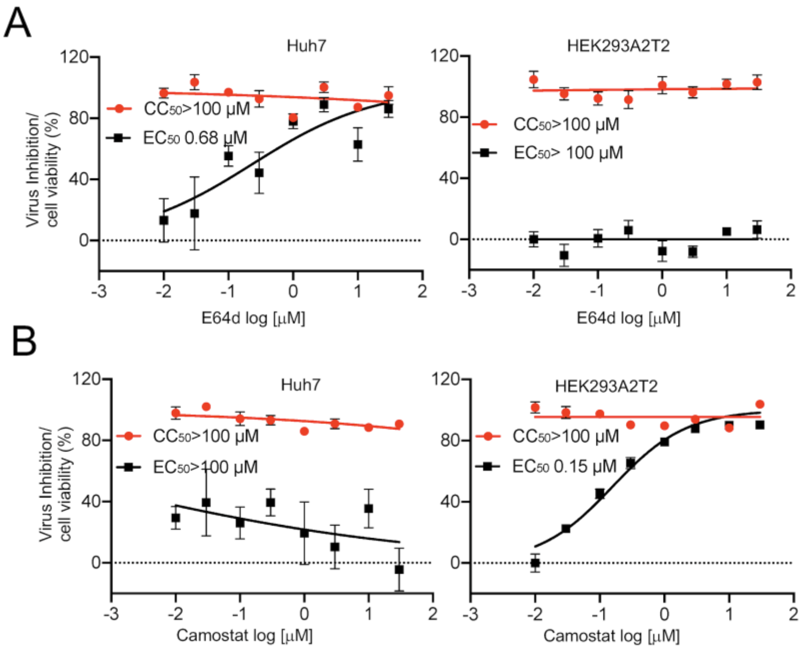

Supplemental Figure 1. Effects of E64d and camostat on plasma membrane and endosomal entry pathway.

Huh7 and HEK293A2T2 cells were infected with VSV based SARS-CoV-2 pseudovirus and treated with multiple E64d **(A)** and camostat **(B)**. At 24 h post-infection, the luciferase activity was measured, and the % virus inhibition and cell viability were determined by normalizing the viral luciferase or cell viability (ATP assay values) with DMSO control. The red and black series represent cell viability and virus inhibition respectively. The data are presented in terms of mean with SEM (n=4) and representative of three independent experiments.

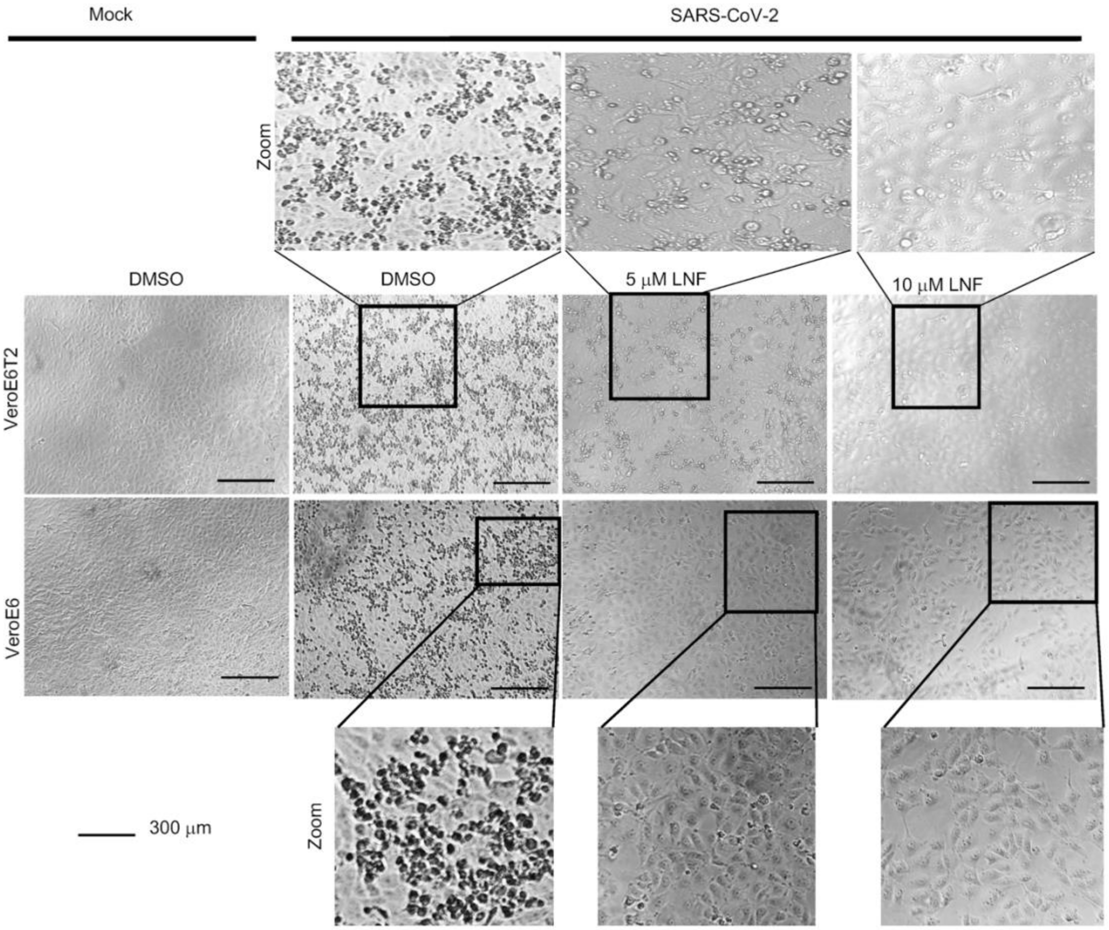

Supplemental Figure 2. Effect of LNF on virus-induced cytopathic effect (CPE).
VeroE6 and Vero-TMPRSS2 cells were infected with SARS-CoV-2 and treated with LNF. At 72h post-infection, cells were analyzed under microscope and CPE was visually analyzed. The images are representation of at least 4 random areas and inset areas are zoomed for better view. This experiment was representative of three independent experiments. Bar scale represents 300 μm.

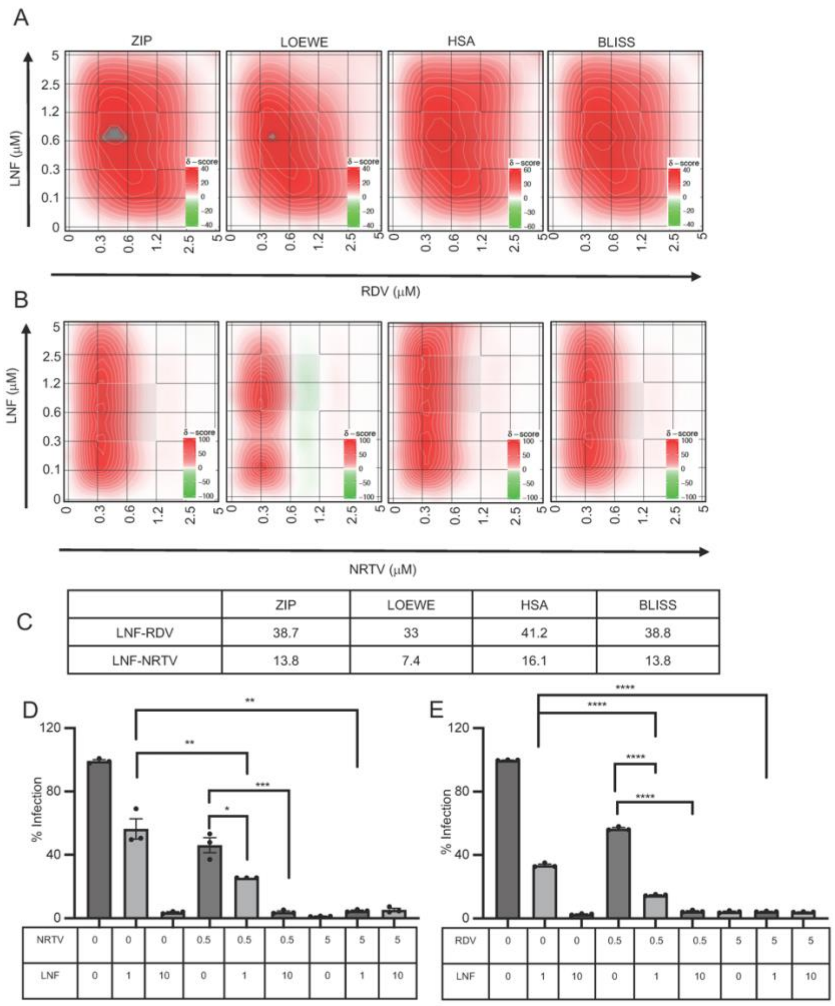

Supplemental Figure 3. Effect of LNF on SARS-CoV-2 variants and LNF-synergy with RDV and NRTV. The SARS-CoV-2-nLuc-infected Calu3 cells were treated with multiple concentrations of LNF-NRTV and LNF-RDV combinations at the time of infection. At 24 h post-infection the luciferase activity was measured and replication relative to DMSO treated control was calculated. **(A, B)** Viral inhibition achieved by drug-combinations of varying concentrations of LNF and RDV (A) or NRTV (B) is analyzed by SynergyFinder. Infected cells were treated with compounds at concentrations ranging from 0-5 μM. Viral infectivity was normalized with the untreated (DMSO) infected cells and percent of inhibition was calculated. Data represent mean values from three independent experiments and contour graphs for ZIP, Loewe, HSA and BLISS synergy are plotted using Synergyfinder. **(C)** The panel summarizes different synergy score statistics for LNF-RDV and LNF-NRTV combination. **(D)** The LNF-NRTV and **(E)** LNF-RDV synergy was tested using omicron BA4.6 variant. The VeroE6 cells were infected with omicron BA4.6 and treated with multiple combinations of the drugs as shown in the table below x-axis. At 24 hpi, viral RNA was purified, and total genome load was measured using qRT-PCR. The graph values are the mean ± SEM of triplicates and represent three independent experiments. The significance was determined my unpaired t-test and the *P* values are depicted as * for *P*<0.05, ** for *P*<0.01, *** *for P*<0.001, and **** for *P*<0.0001.

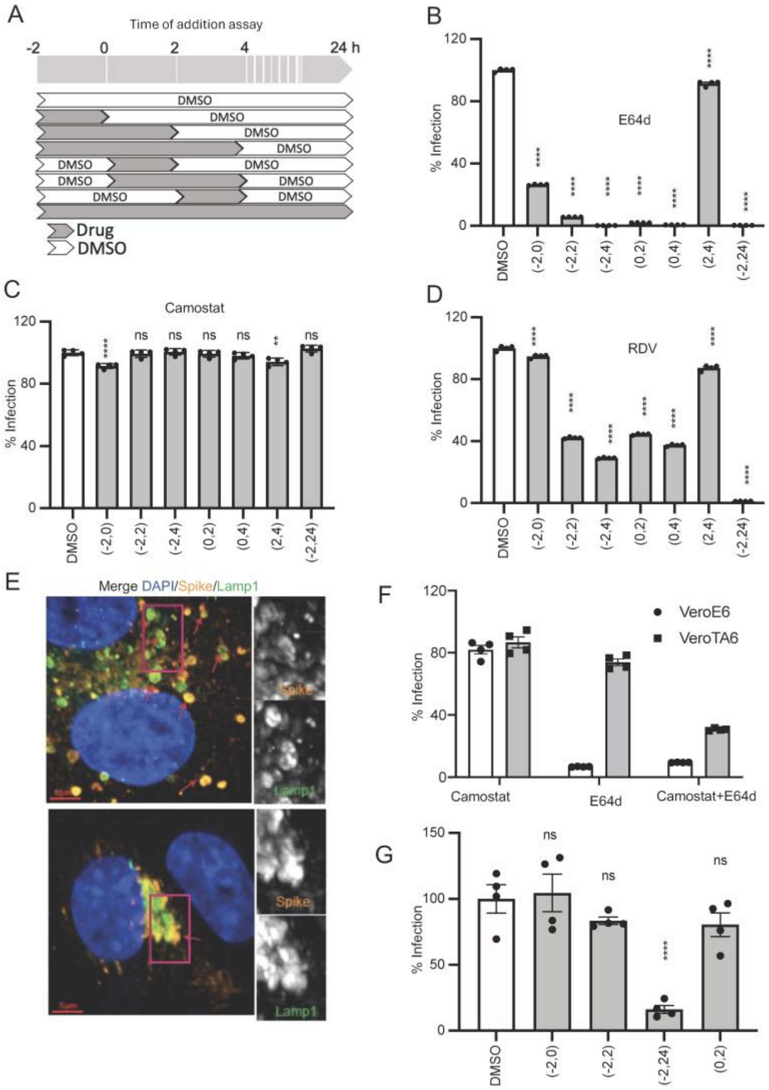

Supplemental Figure 4. Time of addition assay using entry and replication inhibitors.
(A) Schematic of drug treatment plan where solid and empty areas represent the presence and absence of the drug, respectively. The 0 hour represent the time of infection. DMSO was used as control. **(B-D)** The infected cells were treated with 5 μM of E64d (B), camostat (C) and RDV (D) as per the schematic. At 24 h post-infection, the luciferase activity was measured and normalized against untreated control. The viral replication was graphed as percent infection relative to DMSO treated control group. All bars represent mean ± SEM and the figure is a representation of 3 independent experiments. The significance was calculated using one-way ANOVA with Dunnett's test with multiple comparison to the DMSO control, and the P values are depicted as ns for $P>0.05$, ** for $P<0.01$, and **** for $P<0.0001$. **(E)** Cells were infected with virus at 0.1 MOI for 4 hours, fixed, and stained with antibodies against spike protein (red), endolysosomal marker Lamp1 (green), and DAPI (blue). Shown are 3D view examples of infected VeroTA6 (top) and VeroE6 (bottom). Scale bar represents 5μm. The red arrow heads indicate colocalization of spike and Lamp1. The box (magenta) labeled area is enlarged view of stained images. **(F)** VeroE6 and VeroTA6 cells were infected with SARS-CoV-2-nLUC and treated with 10 μM of camostat and

E64d. At 24h post infection, the luciferase activity was measured and normalized to the untreated control. The values are the mean± SEM and the figure is a representation of three independent experiments. **(G)** Calu3 cells were infected with SARS-CoV-2-nLUC and treated with LNF (10 μM) for multiple time duration as shown in x-axis. At 24 hpi nLUC activity was measured and relative infectivity was determined. The data are the mean± SEM and is a representation of two independent experiments. The significance was calculated using one-way ANOVA with Dunnett's test with multiple comparison to the DMSO control, and the *P* values are depicted as ns for $P>0.05$, and *** for $P<0.001$.

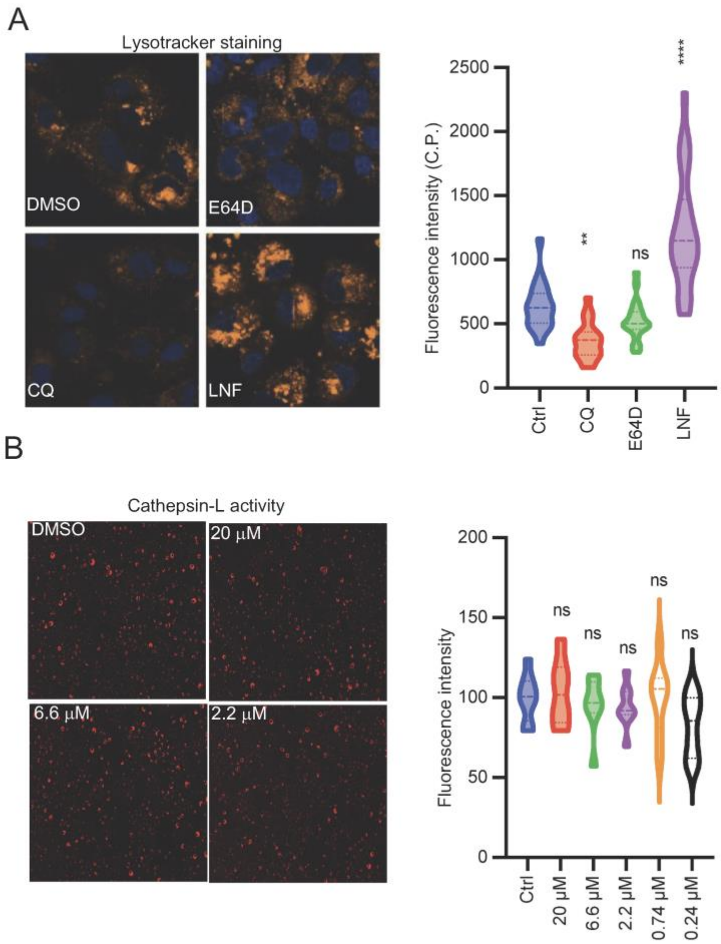

Supplemental Figure 5. LNF increases lysosomal acidification. (A) VeroTA6 cells were seeded in 8-chamopbered slides and treated with 10 μM of chloroquine, E64d and LNF. At 30-minute post treatment, the cells were stained with lysotracker (Thermofisher, L7528) and visualized under the microscope (left). The fluorescence intensity was captured and quantified for each treatment (right). **(B)** For cathepsin-L activity assay, VeroTA6 cells were seeded in 96-well black/clear bottom plates and treated with different concentrations of LNF. At 30-minute post treatment, cathepsin-L activity was measured using cathepsin activity kit (Immunochemistry, 941). Total 8 wells were included for each concentration and 4 random fields per well were imaged using plate scanner. Representative images are shown for each concentration (left). The mean fluorescence intensity (MFI) was measured and quantified using SprectraMax cell imaging system algorithm (right) and graph was plotted using percent MFI for each concentration. For both the panels (A & B), the significance was calculated using one-way ANOVA with Dunnett's test with multiple comparison to the DMSO control, and the *P* values are depicted as ns for $P>0.05$, ** for $P<0.01$, and *** for $P<0.001$.

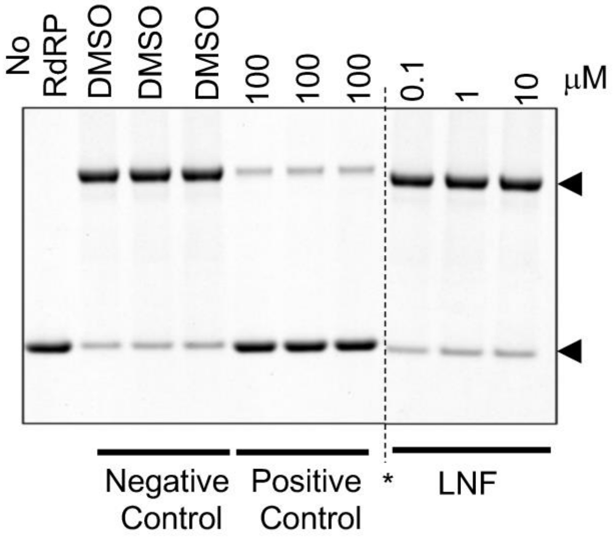

Supplemental Figure 6. SARS-CoV-2 RdRp is not a direct target of LNF. RNA polymerase activity of purified RdRp in the presence of LNF was analyzed as described in the Materials & Methods. No RdRP (lane 1) was used as a negative control for the reaction and 100 μM TEMPOL (lane 5-7) served as a positive control. LNF (lane 8-10) was tested at concentration of 0.1, 1 and 10 μM and the similar equivalent concentration of DMSO (lane 2-4) was used as a vehicle control. The arrowheads indicate the primer (bottom) and primer-extended product (top). * Vertical dashed line represents splicing of gel image.

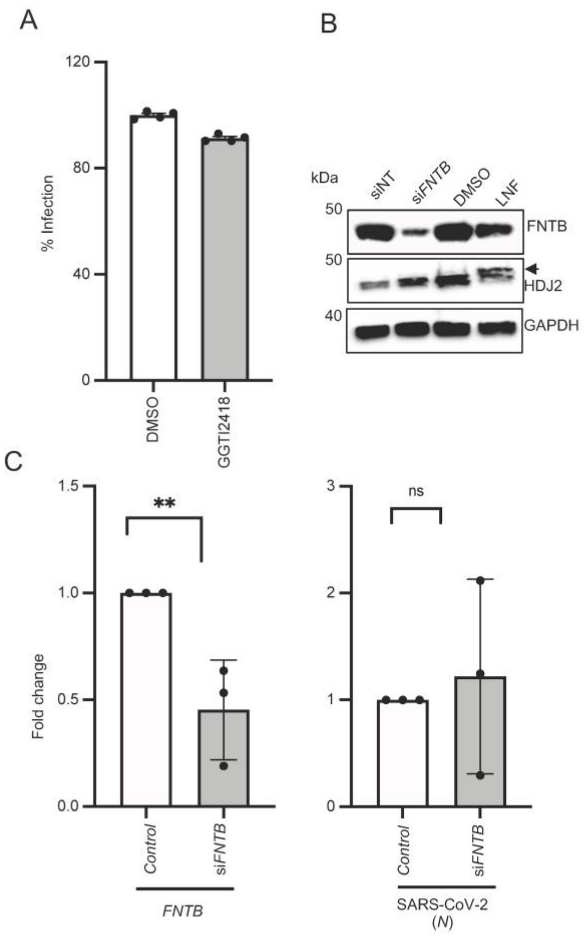

Supplemental Figure 7. Effect of GGTase and FTase on SARS-CoV-2 replication.
(A) VeroE6 cells were infected with SARS-CoV-2-nLuc and treated with 15 μM of GGTI. At 24 h post-infection, nLUC activity was measured and viral replication was quantified relative to DMSO control. **(B)** VeroE6 cells were transfected with si*FNTB* (Dharmacon). At 48 h post-transfection, cells were lysed in RIPA buffer and expression of FNTB, HDJ2 was analyzed by western blot. Arrow indicates shift in HDJ2 mobility, a signature of FTase inhibition. **(C)** VeroE6 cells were transfected with si*FNTB* and siNT. At 24 h post-transfection, cells were infected with SARS-CoV-2 and total RNA was isolated after 24 h of infection. The knockdown efficiency of siRNA was shown by checking the *FNTB*-RNA level relative to that of siNT control as fold change (left). The effect of si*FNTB* was analyzed by determining SARS-Cov-2 genome level (right), which was calculated as fold change relative to control (siNT). The significance was determined by unpaired *t*-test and the *P* values are depicted as ns for $P>0.05$ and ** for $P<0.01$.

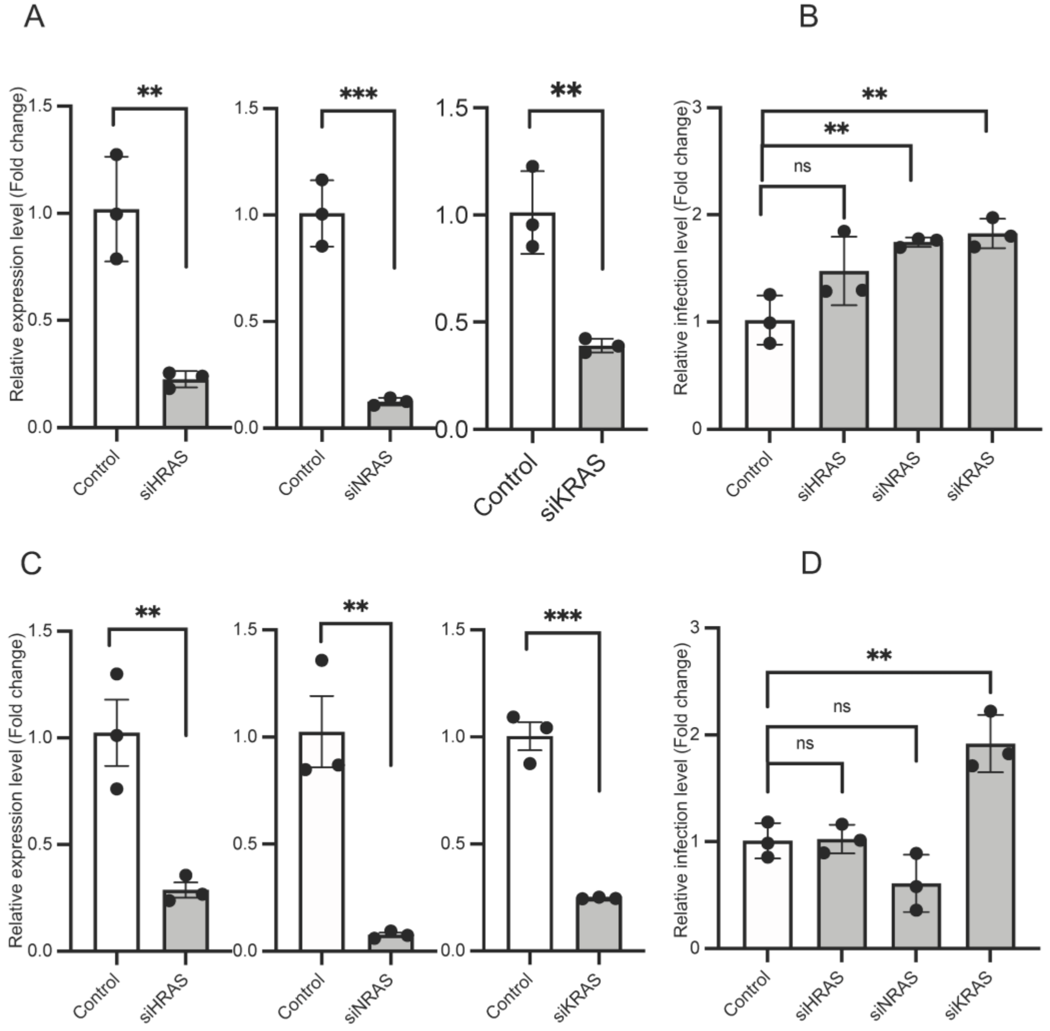

Supplemental Figure 8. Effect of RAS-proteins depletion on SARS-CoV-2 replication.
(A, B) Caco2 cells were transfected with siRNAs specific to *HRAS*, *KRAS* and *NRAS* genes. At 24 h post-transfection, cells were infected with VSV-Spp. At 24 h post-infection, total RNA was isolated, and siRNA efficiency was confirmed by analyzing the relative expression of the target gene transcripts normalized to those of siNT control (A). The level of VSV-Spp infection was determined in RAS-depleted cells using *L*-gene specific primers normalized to those of siNT control and expressed as fold change (B). **(C, D)** Similarly, VeroE6 cells were treated with specific siRNAs and infected with SARS-CoV-2. The siRNA efficiency was then confirmed by analyzing the relative expression of the target gene transcripts (C) and the relative level of SARS-CoV-2 infection was determined using *N*-gene specific primers (D). The relative levels of the RNAs are presented as fold change relative to control (siNT). Each data represent mean ± SEM and the experiment is a representation of triplicates. All the bars show mean values with SEM and representative of three independent experiments. For the panels A & C, the significance was determined by unpaired *t*-test and for panels B & D, one-way ANOVA with Dunnett's test with multiple comparison to the control was used, The *P* values are depicted as ns for $P>0.05$, ** for $P<0.01$, and *** for $P<0.001$.

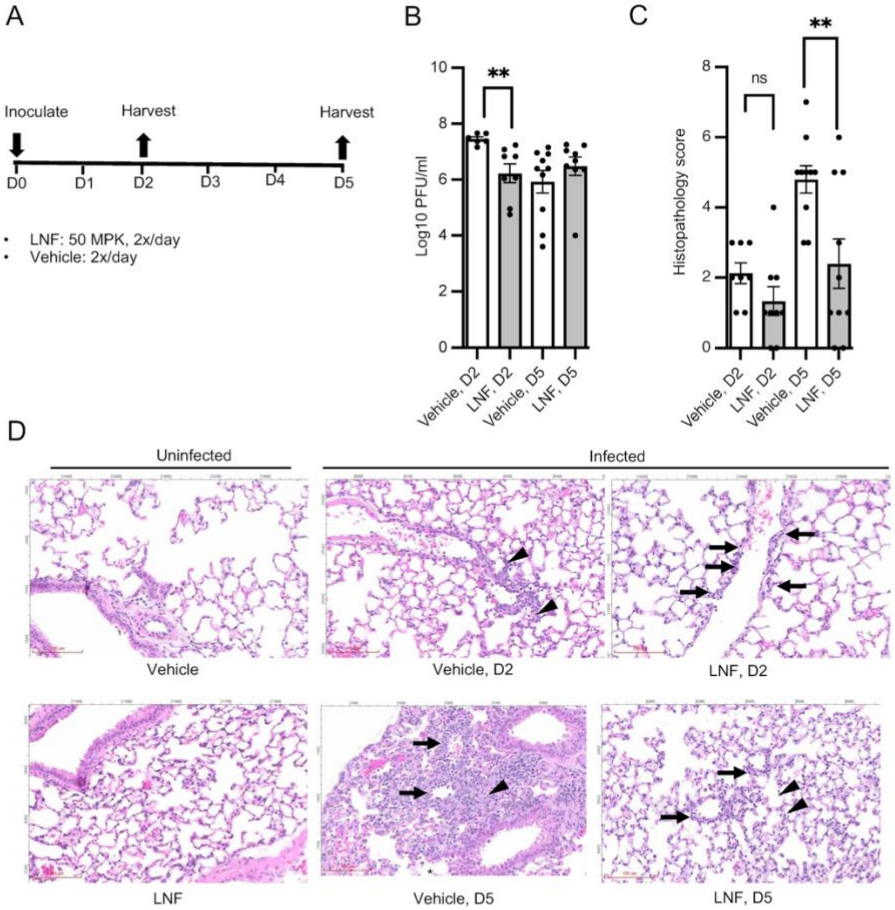

Supplemental Figure 9. Oral efficacy of LNF in animal model. (A) Drug treatment scheme showing how the infected K18-*hACE2* mice were treated with LNF. **(B)** Lung tissue harvested at day 2 and day 5 post treatment were processed as per the process described in the Method section. Values were plotted as log10 PFU/sample, and each bar represents mean, and SEM. **(C)** Tissue sections were analyzed and graded individually (from 0-3) representing the degree of alveolar inflammation as well as degree and frequency of necrosis/hyaline membrane formation and perivascular inflammation. The values were summed to obtain a composite histopathology score. The significance was determined by unpaired t-test with Welch's correction. The adjusted *P* values are depicted as ns for *P*> 0.01 and ** for *P* value 0.01. **(D)** Representative H&E-stained histopathology images of lung from mock-infected (left) and infected animal treated with vehicle (middle) or LNF (right) harvested at day 2 (D2, upper row). And day 5 (D5, lower row). Control animals (vehicle-treated, infected) exhibited lesions, which were characterized by neutrophils and lesser lymphocytes and histiocytic cells present within alveoli and surrounding vessels. In contract, LNF treated animals showed minimum to low signs of inflammation within alveoli and surrounding vessels.
