# Supplementary material for: Repurposing of lonafarnib as a treatment for SARS-CoV-2 infection
Source: JCI Insight. 2025 Jan 9;10(1):e182704. doi: 10.1172/jci.insight.182704 (PMC11721293; doi:10.1172/jci.insight.182704)
Supplement: Unedited blot and gel images [file jciinsight-10-182704-s104.pdf]

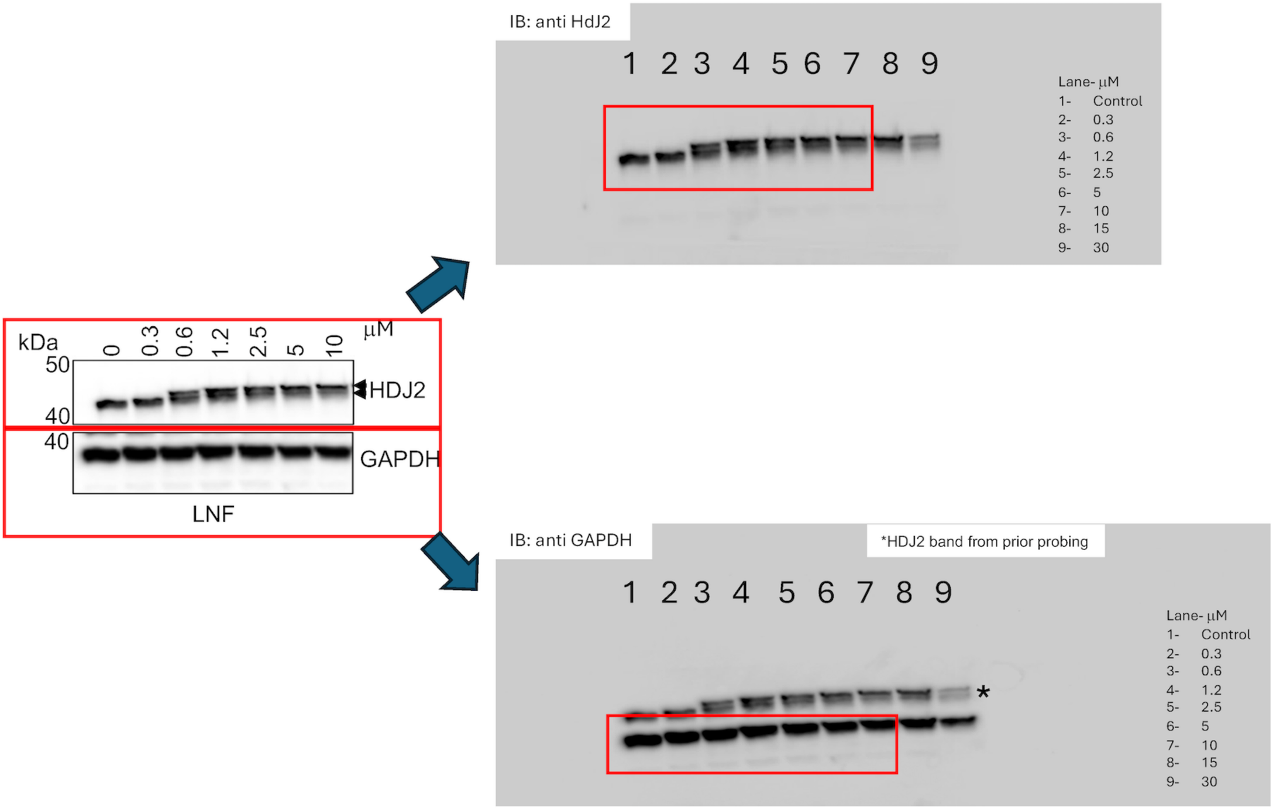

Full unedited gel for Figure 5C, left panel

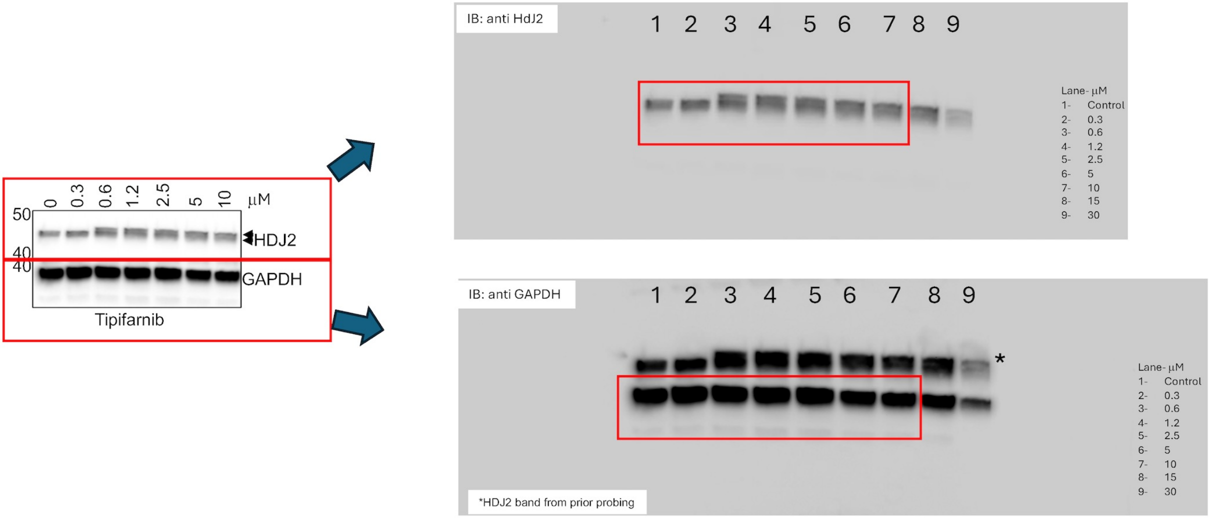

Full unedited gel for Figure 5C, middle panel

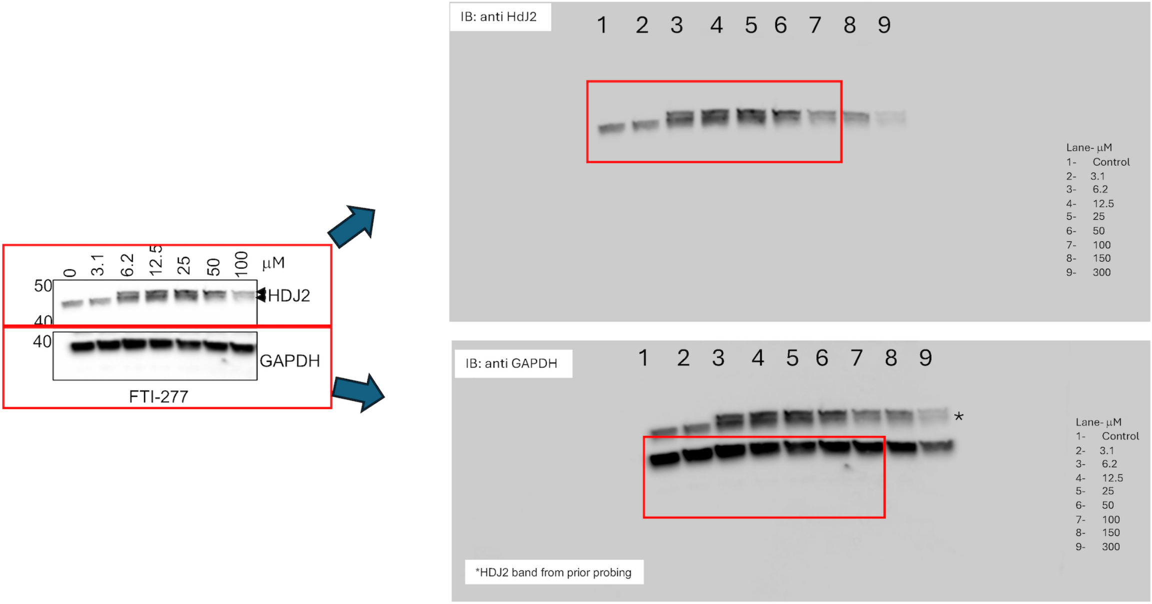

Full unedited gel for Figure 5C, right panel

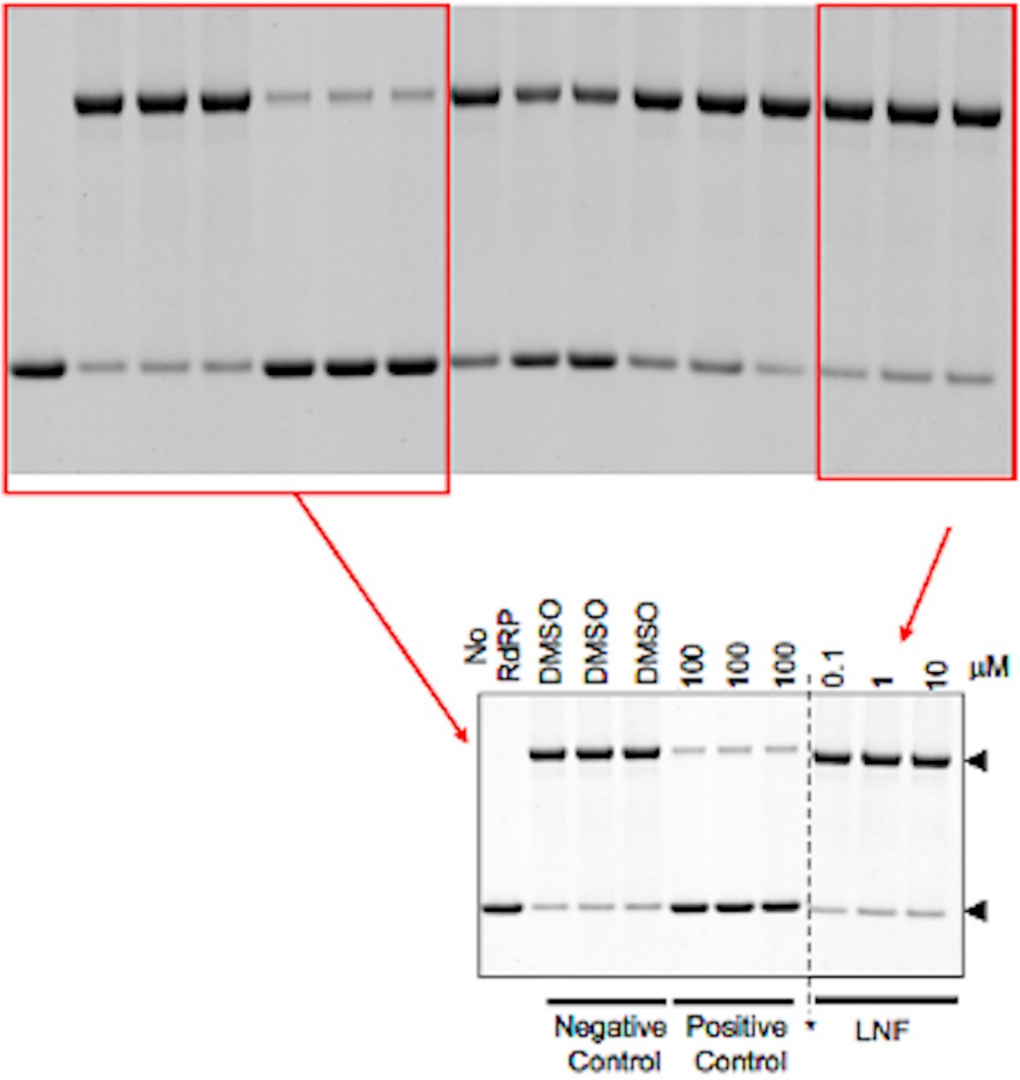

Full unedited gel for Supplemental Figure S6

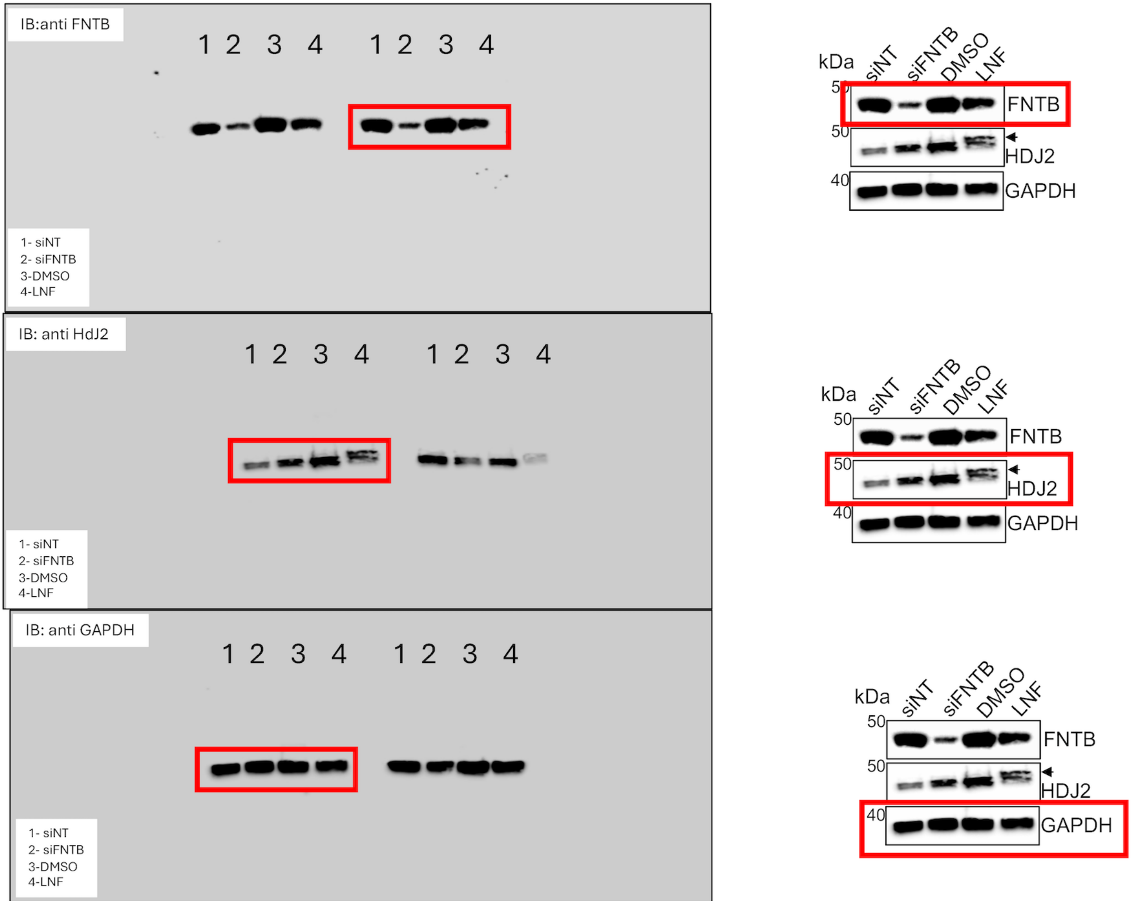

Full unedited gel for Supplemental Figure S7B
